# Restructuring of the epiphytic microbiome and recruitment of algicidal bacteria by *Vallisneria natans* for the suppression of *Microcystis*

**DOI:** 10.3389/fpls.2025.1731742

**Published:** 2026-01-14

**Authors:** Yunni Gao, Ying Wei, Dahai Zeng, Jingxiao Zhang, Jing Dong, Xiaofei Gao, Huatao Yuan, Xuejun Li, Dongru Qiu, Michele Burford

**Affiliations:** 1 College of Fisheries, Henan Normal University, Xinxiang, China; 2 Observation and Research Station on Water Ecosystem in Danjiangkou Reservoir of Henan Province, Nanyang, China; 3 The National Ecological Quality Comprehensive Monitoring Station (Hebi Station), Hebi, China; 4 Institute of Hydrobiology, Chinese Academy of Sciences, Wuhan, China; 5 Australian Rivers Institute, Griffith University, Meadowbrook, QLD, Australia

**Keywords:** algicidal bacteria, epiphytic microbiome, metabolic pathway, *Microcystis*, *Vallisneria natans*

## Abstract

The effective suppression of cyanobacteria by submerged macrophytes is a key mechanism underlying the successful restoration of aquatic vegetation in some eutrophic water bodies. However, the responses and functional roles of epiphytic microorganisms in this process remained largely unclear, limiting a clear understanding of how macrophytes inhibit cyanobacterial growth. In this study we investigated the temporal dynamics of the epiphytic microbiome on *Vallisneria natans* before, during and after exposure to toxic cyanobacterium *Microcystis*, corresponding to three distinct physiological stages of the plant: pre-stress, stress, and recovery. It was observed that the diversity of epiphytic bacteria and eukaryotic algae increased during the stress stage, while that of other eukaryotes, particularly fungi and protozoa, decreased. The complexity and stability of the epiphytic microbiome were enhanced, with bacteria emerging as central hubs in the co-occurrence network in response to *Microcystis* stress. More importantly, a selective enrichment and recruitment of potential algicidal bacteria, particularly *Streptomyces*, *Pseudomonas* and *Chryseobacterium*, occurred on macrophyte surfaces during the stress phase. Their abundance peaked under *Microcystis* stress and returned to baseline levels during the plant recovery phase. Our findings demonstrate that *V. natans* did not function alone, but rather actively recruited and sustained a beneficial microbiome to enhance its suppressive effects on *Microcystis*. This study revealed a previously neglected macrophyte-epiphytic microbiome synergy, providing novel mechanistic insights into how submerged vegetation effectively suppresses harmful cyanobacteria.

## Introduction

Selective suppression of cyanobacteria by submerged macrophytes is one indispensable strategy for their successful restoration in degraded eutrophic waterbodies (Scheffer et al., 2001; Peng et al., 2022; Yang et al., 2023). Allelopathy has been widely regarded as the primary mechanism by which submerged macrophytes inhibit cyanobacteria (Gao et al., 2017; Hilt and Gross, 2008; Nakai et al., 2012). A recent meta-analysis indicates that the contribution of allelopathy surpasses those of shading and nutrient competition (Liu et al., 2024). A large body of research has demonstrated that submerged macrophytes are capable of producing and releasing allelochemicals, such as polyphenols, which directly suppress cyanobacteria in the surrounding environment (Gao et al., 2017; Hilt and Gross, 2008; Jeong et al., 2021; Švanys et al., 2014). The physiological mechanisms likely involve damage to cellular membranes, inhibition of key physiological processes including photosynthesis, and induction of programmed cell death (Zhu et al., 2010; Lu et al., 2017; Li et al., 2021; Ni et al., 2021). However, the trace levels of allelochemicals released by submerged macrophytes appear insufficient to fully account for the observed cyanobacterial inhibition from their donor hosts (Gao et al., 2017). There are estimates of a maximum of 53% of the allelopathic effect being explained by multiple allelochemicals (Nakai et al., 2012). It suggests that additional cyanobacteria-inhibiting mechanisms must exist among submerged macrophytes.

Similar to terrestrial plants, submerged macrophytes harbor a diverse array of epiphytic microorganisms at the epiphytic interface. This complex microbial assemblage includes prokaryotes, such as bacteria, and eukaryotes comprising algae, protozoa, fungi, and others (Perreault and Laforest-Lapointe, 2022; Sohrabi et al., 2023). Compared with other epiphytic microbial communities, the epiphytic bacterial community has been the most extensively studied (Schlechter et al., 2019; Zhen et al., 2020b; Yu et al., 2022; Wang et al., 2024). The responses and functional roles of epiphytic bacteria to various abiotic environmental factors have been investigated more extensively than those related to biotic factors in aquatic ecosystems (Ohore et al., 2021; Geng et al., 2022; Qin et al., 2022; Yang et al., 2025). Epiphytic bacteria have the potential to enhance nitrogen removal, mitigate emissions of greenhouse gases like methane, and reduce the risks posed by heavy metal such as Mn, Cr and As in aquatic environments (Zhen et al., 2020a; Geng et al., 2022; Lu et al., 2023; Deng et al., 2024; Jiang et al., 2024; Sun et al., 2024). Previous studies have revealed that *Microcystis* cells, extracts, exudates and cyanotoxins can alter the composition, abundance and diversity of the phyllospheric bacterial communities associated with submerged macrophytes (Jiang et al., 2019; Li et al., 2020; Gao et al., 2022). But our understanding of how other epiphytic microorganisms respond to interactions between submerged macrophytes and cyanobacteria remains limited.

Our recent experiment demonstrated that the *Microcystis*-inhibition rate of crude plant extracts from the submerged macrophyte *Myriophyllum* sp*icatum* decreased significantly from 85.6% to 6.6% following artificial removal of epiphytic microorganisms. Through high-throughput sequencing of 16S and 18S rRNA gene amplicons combined with non-targeted metabolomics analysis, multiple potentially algicidal microorganisms and associated metabolites were identified in the epiphytic biofilms (Wei et al., 2024). These results suggest that epiphytic microorganisms may play a critical role in the plant host’s ability to inhibit *Microcystis*. But we need more direct evidence to reveal the role of epiphytic microorganisms during the process of real-time interaction between submerged macrophytes and harmful cyanobacteria.

It has long been recognized that terrestrial plants can release “cry for help” signals through chemical communication to recruit beneficial microorganisms to the rhizosphere or phyllosphere (Tsai et al., 2025; Zeng et al., 2025). This mechanism has been extensively validated in model plants such as *Arabidopsis thaliana* and crops including wheat, corn and rice, particularly in the context of defense against pathogens and pests (Berendsen et al., 2018; Rizaludin et al., 2021; Danso Ofori et al., 2024), and its potential for facilitating the degradation of soil pollutants through the plant microbiome has also been demonstrated (Rolli et al., 2021). For submerged macrophytes, analysis of epiphytic bacterial dynamics during periods of high ammonia nitrogen stress and subsequent recovery revealed that *V. natans* actively recruited beneficial bacteria, restructured its phyllospheric microbial community, and thereby enhanced its resistance to ammonia nitrogen stress (Hu et al., 2023). However, it remains unclear whether submerged macrophytes can recruit specific beneficial bacteria, such as cyanobactericidal strains, to strengthen their inhibitory effects on cyanobacteria.

To explore the response dynamics of epiphytic microbial community at the phyllosphere to the inhibition process of submerged macrophytes on cyanobacteria and the potential functions, one of the most commonly used submerged macrophytes for ecological restoration, *V. natans*, and the most typical bloom-dominated cyanobacterial species- *Microcystis* was selected, to investigate the response dynamics of epiphytic microbial community during the entire inhibition process of *V. natans* on *Microcystis*, including three characteristic phases of pre-stress, stress period and recovery period. The study aims to verify the following hypotheses: (1) The response dynamics of epiphytic bacteria and eukaryotes during the inhibition of *Microcystis* by host submerged macrophyte *V. natans* may be different. (2) Submerged macrophyte *V. natans* might recruit beneficial microorganisms to help inhibit *Microcystis*.

## Materials and methods

### Cultivation of *Microcystis* and *V. natans*

Fresh macrophyte, *V. natans* seedlings were collected from our aquatic plant breeding tanks containing 10 cm-deep aquarium black soil substrates (Anubias, Japan). These tanks have been used to cultivate *V. natans* since 2020. The original *V. natans* plants were transplanted from Honghu Lake (N29.827°, E113.476°) in Hubei Province, China. One microcystin (MC)-producing *Microcystis* strain (FACHB-915) was obtained from the Freshwater Algae Culture Collection at the Institute of Hydrobiology, the Chinese Academy of Sciences.

The selected plant seedlings (12 ± 1 cm high) and *Microcystis* cells were pre-cultured separately. *Microcystis* was cultured in 1/10 diluted BG-11 medium in controlled conditions with a temperature of 22 ± 3 °C, a 12:12 light: dark cycle, and a light intensity of 25 μmol photon (PAR) m^−2^ s^−1^. The *Microcystis* cells in the exponential growth phase were used for experiments.

### Experiment design

**Figure 1:**
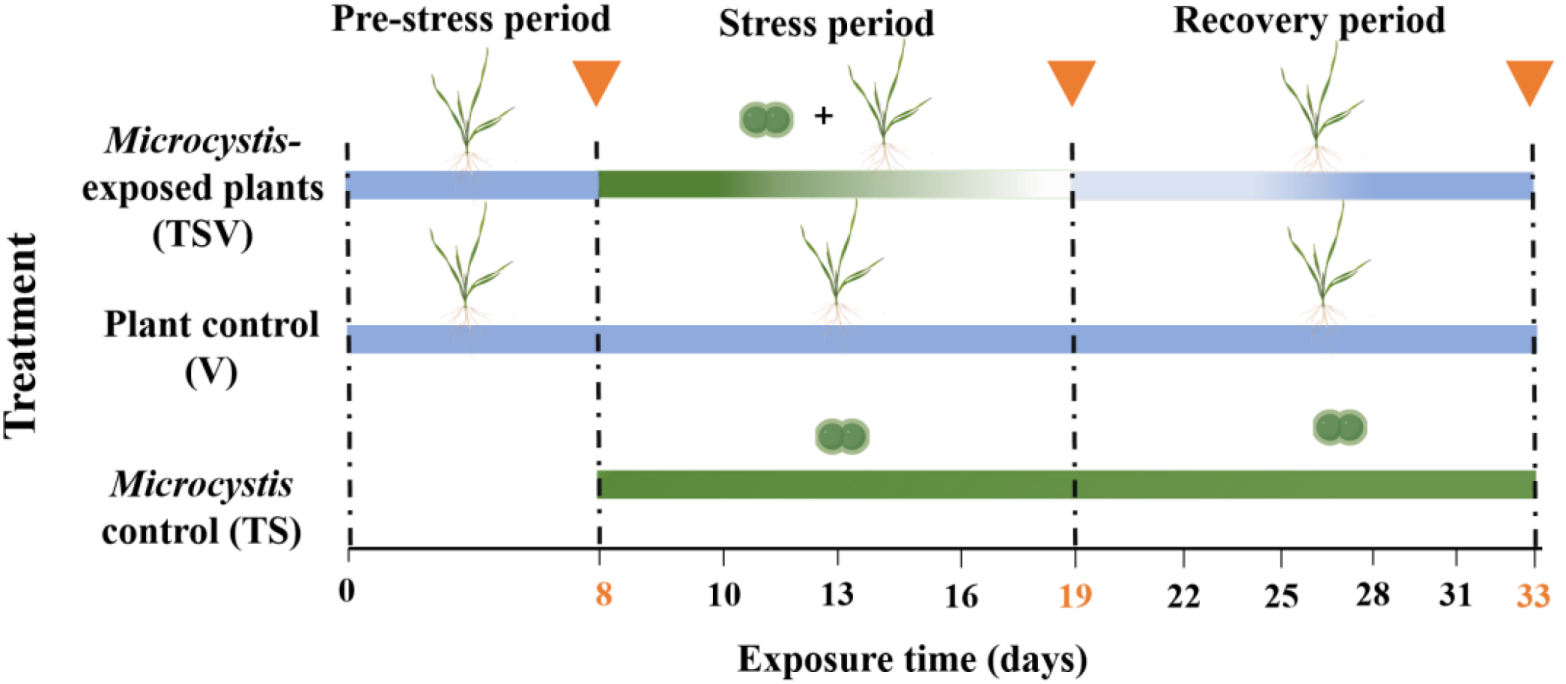
Schematic diagram of the experimental setup. The inverted triangles represent the end of each phase.

**Figure 2:**
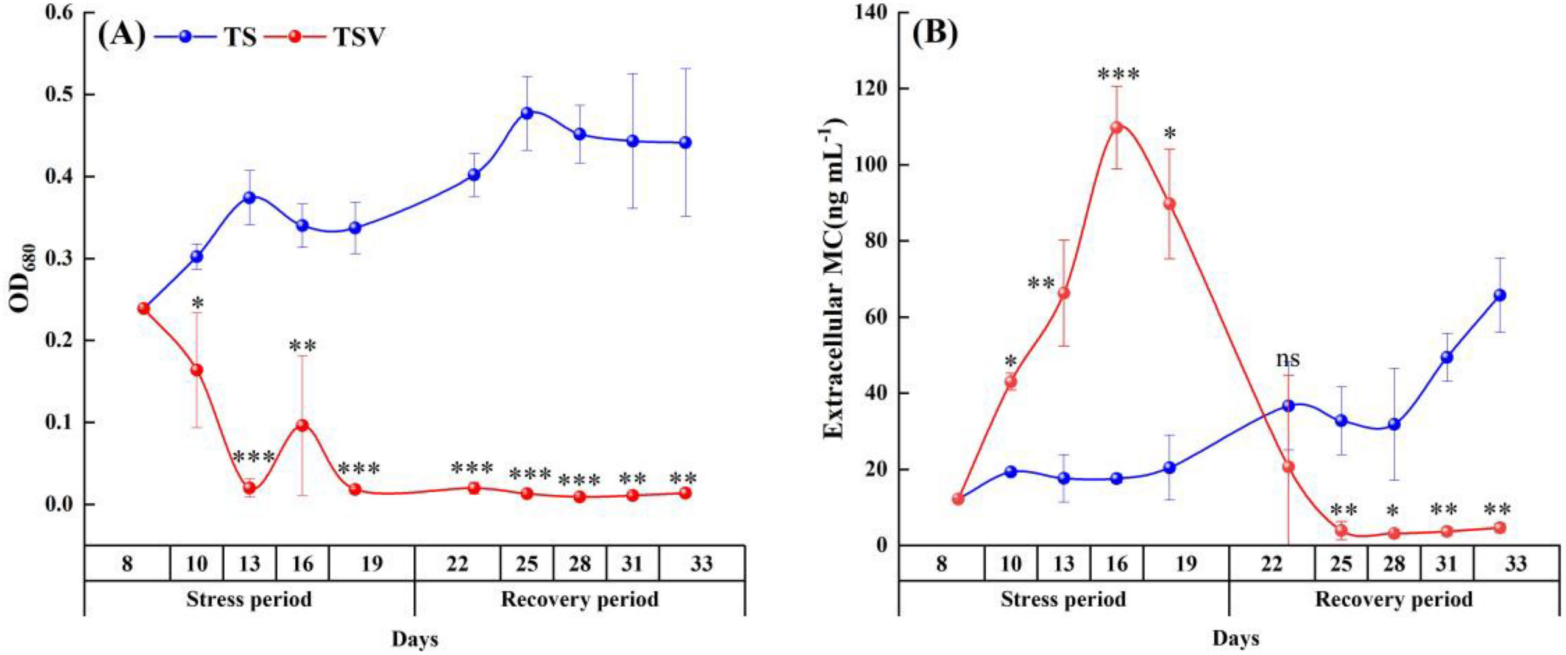
The optical density curves of *Microcystis* culture **(A)** and the extracellular microcystin (MC) concentrations **(B)** in the TS and TSV groups. Data are means ± standard deviation analyzed from four parallel samples. *, ** and *** indicate significant differences between the two groups at *p* < 0.05, *p* < 0.01 and *p* < 0.001, respectively.

**Figure 3:**
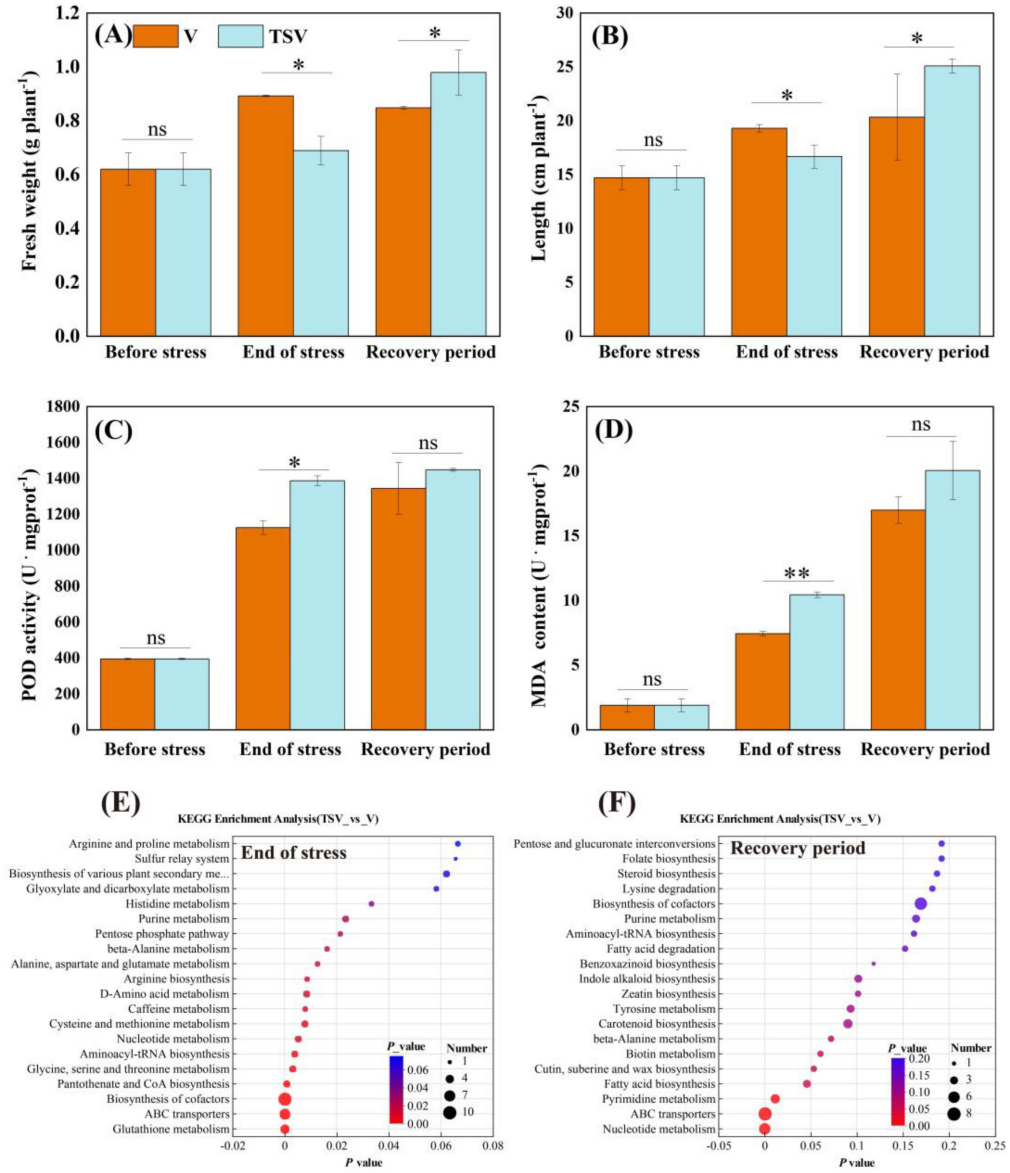
Fresh weight **(A)**, plant length **(B)**, peroxidase (POD) activity **(C)**, and malondialdehyde (MDA) content **(D)** of *V. natans*, as well as KEGG enrichment analysis of plant metabolites in both V and TSV groups at the end of the stress period **(E)** and recovery period **(F)**. Data are means ± standard deviation analyzed from three parallel samples. * and ** indicate significant differences between the two groups at the same time at *p* < 0.05 and *p* < 0.01. ns indicates no significant differences.

The indoor microcosm experiments included three groups: one treated group where *V. natans* is exposed to *Microcystis* (TSV), one plant control group with only a monoculture of *V. natans* (V), and one *Microcystis* control group with only a monoculture of *Microcystis* (TS) (**Figure 1**). In the TSV group, *V. natans* was initially cultivated at a fresh weight of 2.1 ± 0.06 g L^-1^. The experimental process for this group consisted of three periods: a pre-stress period (0–7 days) for macrophyte acclimation, a stress period initiated on day 8 by the introduction of *Microcystis* at an initial cell density of 3.94 ± 0.32 × 10^6^ cells mL^-1^, and a subsequent recovery period after the suppression of *Microcystis* cells. The entire experiment lasted 33 days. On day 14, when *Microcystis* cells were no longer detectable, a second addition of *Microcystis* cells was introduced into the TSV group. Based on the integrated analysis of *Microcystis* cell density (**Figure 2A**), and the corresponding growth, physiological and metabolic responses of *V. natans* (**Figure 3**), the experimental timeline was divided in to a stress phase (days 8 to 19) and a recovery phase (day 20 onward). The stress phase was characterized by the coexistence of *Microcystis* and *V. natans*, accompanied by a gradual decline in *Microcystis* cell density. By the end of this phase, *V. natans* exhibited significant growth inhibition, elevated oxidative stress, and pronounced disruption in metabolic pathways. In contrast, the recovery phase began following the complete suppression of *Microcystis*, during which *V. natans* transitioned to a monoculture-like state. Throughout this phase, the plants showed marked growth recovery, alleviation of oxidative damage, and restoration of metabolic homeostasis.

The V and TSV groups each consisted of 12 replicates, whereas the TS group had 6 replicates. There was a total of 30 beakers (9 cm in diameter and 15 cm in height) with a 2 cm layer of black soil substrate (Anubias, Japan). The culture conditions during the stress period were the same as those during the preculture period. Three out of four replicates from the V and TSV groups were collected on days 7, 19 and 33 to harvest plants and phyllosphere samples. This collection enabled the investigation of plant and epiphytic microbial responses at three stages. In the TSV and TS groups, four replicates of *Microcystis* samples were collected every two days after inoculation to monitor temporal variation in cell density and extracellular microcystin concentrations. To maintain consistent experimental conditions and compensate for evaporation, an equal volume of 1/10 BG11 medium was added to each treatment group after each sampling.

### Measurement of *Microcystis* growth and microcystin concentrations

A 5 mL of *Microcystis* samples was collected to determine the optical density at a wavelength of 680 nm (OD_680_) by using a spectrophotometer (SPECORD210Plus, Jena, Germany). Another 1 mL of *Microcystis* samples was centrifuged at 10,000×g for 15 minutes. The supernatant was collected and diluted for the determination of extracellular microcystin (MC) concentration using an MC-LR ELISA kit (Institute of Hydrobiology, CAS). It has a detection sensitivity of 0.1 μg L^−1^.

### Determination of plant growth, physiology and metabolites

Fresh weight (FW) and leaf length were measured to observe plant biomass changes. Non-targeted metabolomics were employed to measure the changes of metabolites and metabolic process of *V. natans* in V and TSV groups. A subsample of fresh plant shoots (200 mg) was homogenized with liquid nitrogen for the determination of peroxidase (POD) and malondialdehyde (MDA) with assay kits from Nanjing Jiancheng Company, China (Gao et al., 2022). Exactly 50 mg of plant shoots were placed into a 2 mL grinding tube and extracted with 0.5 mL of methanol-aqueous solution. The detailed pretreatment and subsequent untargeted metabolomics analysis were the same as those in previous studies (Li et al., 2024). Raw metabolomics data have been uploaded to the BIG Submission under the project ID PRJCA053329.

### High-throughput sequencing of 16S and 18S rRNA gene of epiphytic microbiomes

An accurate weight of 1.0 g of plant leaves was collected and transferred to a sterile centrifuge tube containing 50 mL of 0.1 mol L^-1^ PBS (pH 7.0). The tube was then placed on a shaker platform and agitated at 180 rpm for 20 minutes, vortexed for 1 min. This process was repeated twice, and the wash fluids obtained from both repetitions were collected to form suspensions containing epiphytic microbes. Subsequently, 100 mL of suspensions were filtered using a 0.22 μm cellulose acetate filter membrane, and the filter membrane was collected and placed in a 15 mL sterile centrifuge tube, then stored in a -80 °C freezer for further DNA extraction and analysis of epiphytic microbial communities (Gao et al., 2022; Wei et al., 2024) The bacterial V3–V4 hypervariable region of the 16S rRNA genes were amplified by PCR using primers 341 F(CCTAYGGGRBGCASCAG) and 806R (GGACTACNNGGGTATCTAAT). The 18S rRNA gene was amplified using the primers SSU0817F (TTAGCATGGAATAATRRAATAGGA) and 1196R (TCTGGACCTGGTGA GTTTCC). The data were analyzed through the free online platform of major bio cloud platform (Ren et al., 2022). All obtained raw sequence datasets have been uploaded to the NCBI Sequence Read Archive (SRA) with the accession number PRJNA1377903 and PRJNA1377893.

### Statistical analysis

All data were analyzed with Microsoft Excel and SPSS 22.0. The *p*-value < 0.05 was considered statistically significant. Origin 2023 and GraphPad Prism software were used for data visualization and graph processing. Principal Coordinate Analysis (PCoA) was employed to visualize the differences in microbial communities based on the Bray-Curtis distance matrix. KEGG pathway enrichment analysis was performed using Python software (version: 1.0.0). The dominant microbial network was visualized utilizing the “reshape” package within R and Gephi software. The correlation between dominant microbial genera was analyzed using Spearman’s correlation coefficient. The normalized stochasticity ratio (NST) was calculated using the R package to quantify the relative contributions of stochastic and deterministic processes to microbial community assembly, and correct sampling bias through the Modified Stochasticity Ratio (MST). Deterministic process dominated the community assembly if the MST value is less than 0.5, otherwise, stochastic process dominated (Ning et al., 2019).

To explore the relationship between algicidal bacteria and metabolites, we utilized random forest regression analysis. All analyses were carried out using the “random forest” package in R software (version 4.5.1). For each metabolite, its concentration was taken as the response variable, and the abundance data of algicidal bacteria were used as the predictor variables to construct the model. Considering the limited sample size (n = 12), in order to make full use of the data for feature importance assessment, we adopted a full - dataset modeling approach. The model parameters were set as follows: the number of decision trees was set to 1000, and the number of variables randomly considered at each split was set to one third of the total number of features (i.e., 7). To evaluate the goodness of fit of the model to the training data, we computed the coefficient of determination (R²) and the root mean square error (RMSE). To assess the overall statistical significance of the model, we conducted a permutation test: by randomly shuffling the response variable (metabolite concentration) 100 times, reconstructing the model each time and calculating the R² value, a null distribution was established. The actually observed R² value was compared with the null distribution to compute the *p* value.

## Results

### Temporal changes of *Microcystis* growth and extracellular microcystins

The cell density of *Microcystis*, as indicated by OD_680_ values, exhibited a marked decline following the initial introduction to the macrophyte (TSV treatment) on day 8 and after the subsequent introduction on day 14. From day 19 onwards, the OD_680_ value the water containing *Microcystis* decreased to 0.01 and remained at this low level (**Figure 2A**). In contrast, the OD_680_ values in the TS group (Mono-culture of *Microcystis* cells as control) increased from 0.24 on day 8 to 0.44 on day 33.

Despite the inhibition of *Microcystis* cells, the extracellular MC concentrations continued to increase, reaching the maximum value during the late stage of stress phase in the TSV group. This concentration was 5.5 times that of the control group during the same period. Subsequently, it gradually decreased and dropped to a quarter of the initial concentration on the 25th day, remaining at this low level. In the TS group, the extracellular MC concentrations gradually increased and reached 5.8 times the initial concentration by the end of the experiment (**Figure 2B**).

### Growth and physiological metabolic response of *V. natans*

The growth status of *V. natans* in the TSV group, as indicated by the fresh weight of plant tissues and length of plants, was significantly lower in the treatment with *Microcystis* (*p* < 0.05). However, during the recovery period following the decrease of *Microcystis*, the biomass and length of *V. natans* was significantly higher compared to the V group, with plants only (*p* < 0.05, **Figures 3A, B**). During the stress period, *V. natans* in the TSV group exhibited significantly stronger oxidative stress than those in the V group, as evidenced by a marked increase in peroxidase (POD) activity and malondialdehyde (MDA) concentration (*p* < 0.05). In the recovery period, both POD activity and MDA levels remained elevated relative to the control, although the differences were no longer statistically significant (**Figures 3C, D**).

An enrichment analysis of the KEGG pathways, based on the non-target metabolomics of *V. natans*, demonstrated that 16 metabolic pathways of the plants that were altered during the stress period in the TSV group compared to those in the V group. This included glutathione metabolism, ABC transporters, the biosynthesis of cofactors, Poantothenate and CoA biosynthesis, as well as glycine, serine, and threonine metabolism (**Figure 3E**). During the subsequent recovery period, the number of metabolic pathways decreased, to four, including nucleotide metabolism, ABC transporters, pyrimidine metabolism, and fatty acid biosynthesis, all of which exhibited significant differences between the TSV and V groups (**Figure 3F**).

### Temporal changes in the diversity and structure of epiphytic microbiomes

**Figure 4:**
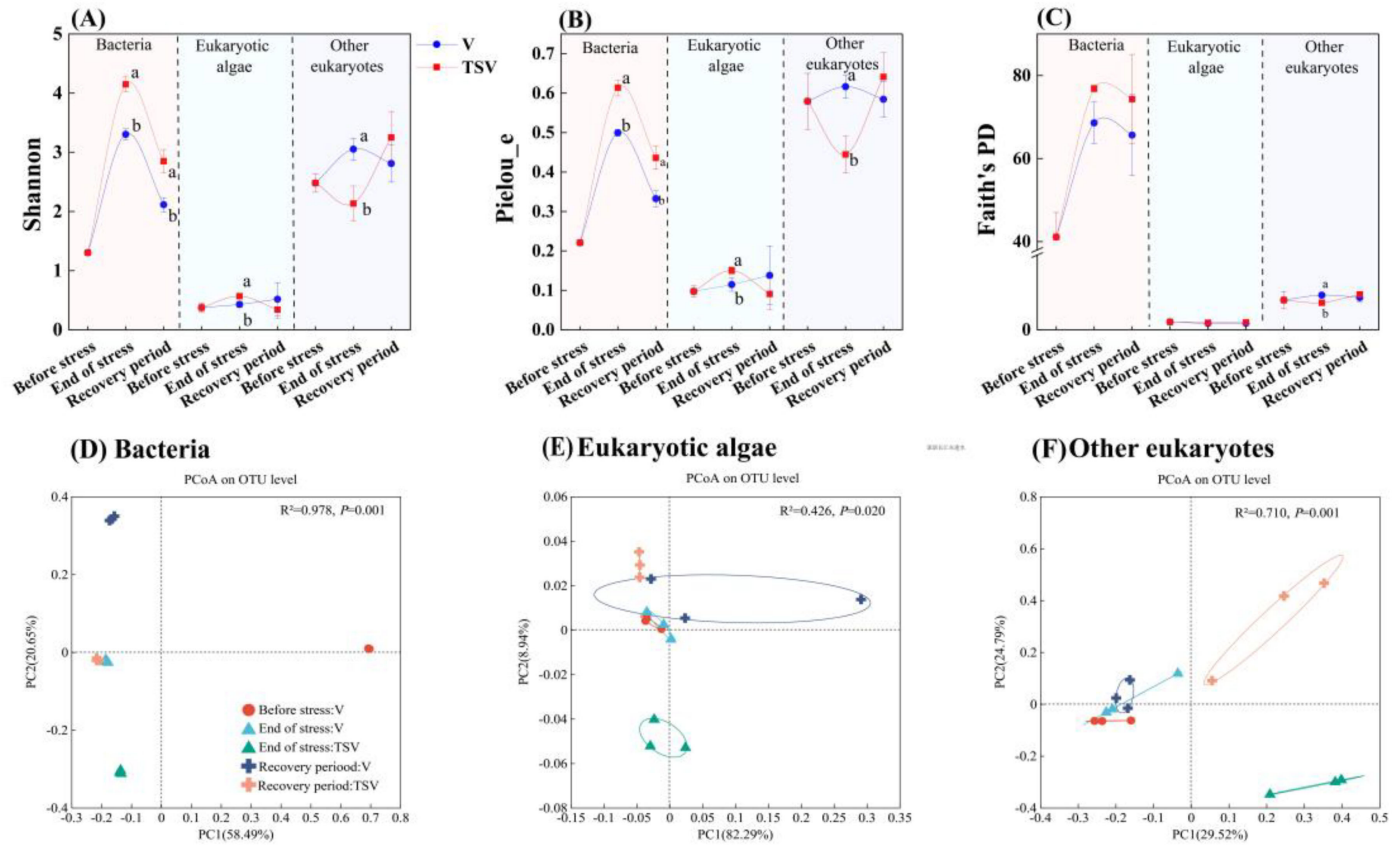
Diversity indices of epiphytic microbiomes during the experimental period. Temporal changes in the Shannon-Wiener diversity index (Shannon, **(A)**), Pielou evenness index (Pielou_e, **(B)**), and Faith’s Phylogenetic diversity index (PD, **(C)**) of the epiphytic bacteria, eukaryotic algae and other eukaryotes. Different letters indicate statistically significant differences between the V and TSV groups within the same period. Data are shown as mean ± standard error (S.E.), with n = 3 biological replicates. Principal coordinates analysis (PCoA) based on Bray–Curtis dissimilarity metrics was performed to assess community composition of epiphytic bacteria **(D)**, eukaryotic algae **(E)** and other eukaryotes **(F)** across different sampling time points and experimental groups.

The alpha-diversity of epiphytic bacteria and eukaryotic algae, as measured by the Shannon and Pielou_e indices, increased during the stress period following the introduction of *Microcystis* cells, and subsequently decreased during the recovery period in the TSV group. However, the Shannon and Pielou_e indices of other eukaryotes mainly including fungi and protozoa in the TSV group decreased significantly during the stress period (*p* < 0.05), subsequently increasing to levels exceeding those in the V group (**Figures 4A, B**). The variation in the Faith’s Phylogenetic diversity index exhibited similar trends as the Shannon and Pielou’s evenness indices, but the differences between the TSV and V groups were not pronounced (**Figure 4C**).

The PCoA demonstrated obvious dissimilarity of the community composition of all epiphytic microbiomes between the TSV and V groups, as well as between the stress period and recovery period in the TSV group. Among the three groups, the temporal variation of epiphytic bacteria community was much bigger than that of eukaryotes, including algae, in the V group (**Figures 4D–F**).

The phyla Proteobacteria, Actinobacteriota and Bacteroidota dominated the epiphytic bacteria of *V. natans* (**Supplementary Figure S1A**). At the genus level, *Pseudomonas* (0.08%~76.06%), *Phreatobacter* (0.06%~19.96%), *Reyranella* (0.04%~9.54%), and *Streptomyces* (0.52%~9.87%) were dominant. The relative abundance of *Reyranella* and *Streptomyces* in the TSV group increased after the introduction of *Microcystis* cells, and was much higher than that in the V group in the same period (**Supplementary Figure S1D**). The attached eukaryotic algae were dominated by diatoms, with a relative abundance ranging from 99.1% to 99.6% (**Supplementary Figure S1B**). The relative abundance of the genera, *Cocconeis* and *Sellaphora* exceeded 80% in both the TSV and V groups, showing no significant differences between the two groups (**Supplementary Figure S1E**).

Other eukaryotes were predominately composed fungi, i.e., Ascomycota and Aphelidea, and protozoa, i.e, Ciliophora (**Supplementary Figure S1C**). The relative abundance of Ascomycota, dominated by the genus *Thysanophora*, decreased substantially in the TSV group compared to that in the V group during the stress and recovery period. However, the relative abundance of the genus *Paraphelidium* belonging to Aphelidea, increased to 54.88% in the TSV group at the end of the stress. The genus *Stentor* was the most dominant within Ciliophora in the V group, but its relative abundance in the TSV group decreased markedly (**Supplementary Figure S1F**).

### Community assembly process and co-occurrence network of epiphytic microbiomes

The modified stochasticity ratio (MST) analysis was applied to reveal the community assembly process of the epiphytic microbiomes (**Supplementary Figure S2**). With the exception of the other epiphytic eukaryotes in the V group, the MST values for epiphytic microbiomes in the V and TSV groups were lower than 0.5, indicating the dominance of the deterministic process (**Supplementary Figures S2A–C**). The deterministic process contributed 94%, 89% and 86% to the community assembly process of epiphytic bacteria, eukaryotic algae and other eukaryotes in the TSV group (**Supplementary Figures S2D–F**). For other eukaryotes, the stochastic process was dominant with the contribution ratio of 78% in the V group (**Supplementary Figures S2C, F**).

**Figure 5:**
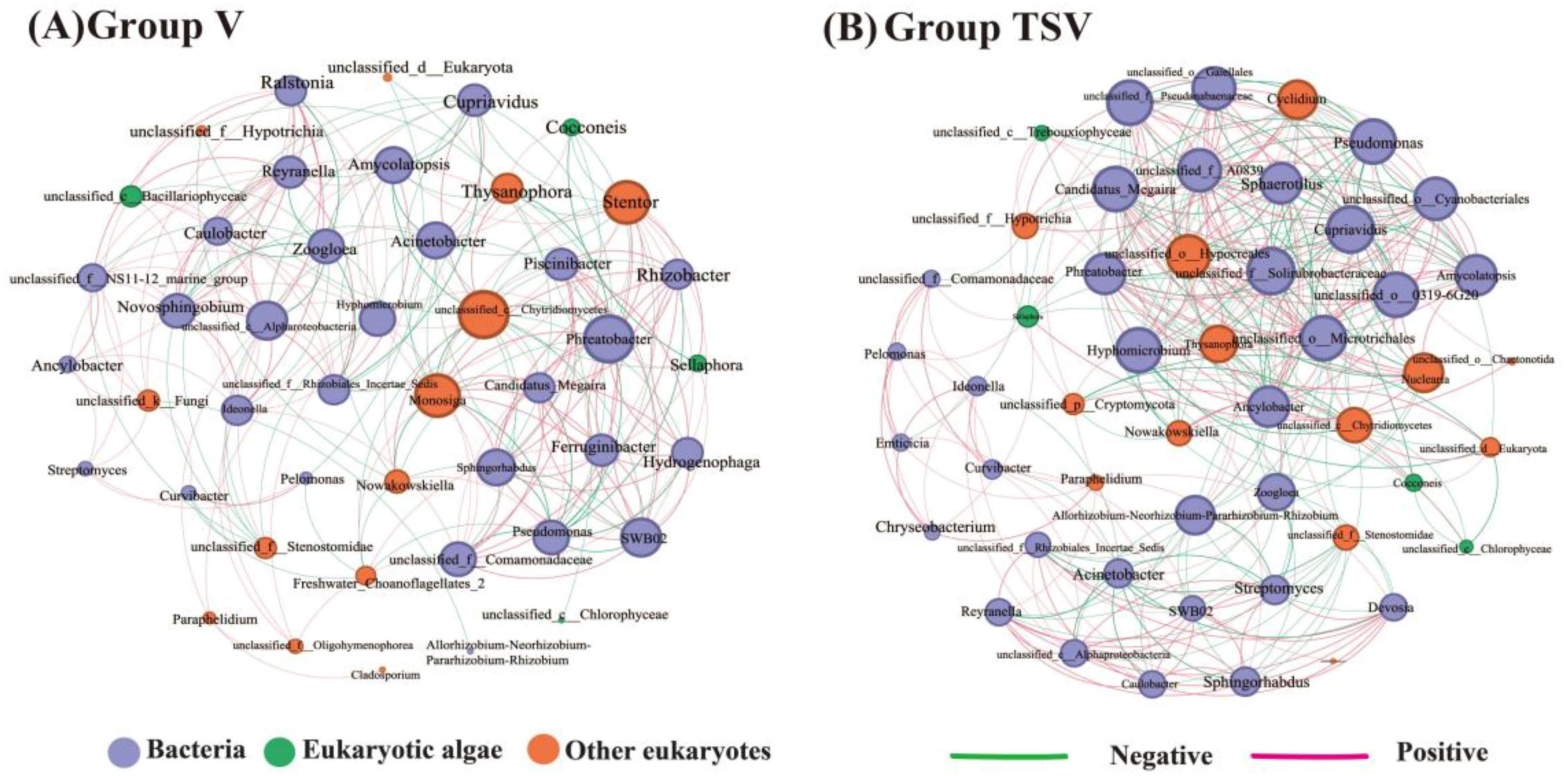
Co-occurrence network analysis of dominant epiphytic microbial genera (>0.1% relative abundance) in groups V **(A)** and TSV **(B)** at three stages. Each node represents one genus and different node colors indicate different domains. The node diameter increased with the number of direct neighbors. Edges represent Spearman’s correlation relationships. Only strong and significant correlations (Spearman’s relationship>0.6, *p* < 0.05) are shown. The red and the green lines indicate positive and negative correlations, respectively. The more lines, the closer the relationship between the species and other species.

To explore the interactions among epiphytic bacteria, eukaryotic algae and other eukaryotes within the V and TSV groups across three stages, co-occurrence networks were constructed using the dominant genera with the relative abundance >0.1% from each microbial group. The modularity values were higher than 0.4 in both groups, indicating the networks of epiphytic microbes have obvious modular structures with intensely connected network nodes. The network properties were altered, with an increase in total nodes and edges, as well as network density and modularity in the TSV group. This suggests that the complexity of the epiphytic microbial community was enhanced and stronger interactions among epiphytic microbes occurred when inhibiting *Microcystis* cells (**Supplementary Table S1**, **Figure 5B**). The hub microorganisms (nodes highly connected to other members in a module) in the TSV group belonged to the bacteria, but were not same as the dominant ones in the V group. The contribution of epiphytic bacteria in the TSV group accounted for up to 65.31%, highlighting the significant roles of epiphytic bacteria in the *Microcystis*-inhibition process by host plants.

Network analysis identified significantly more hub taxa (weighted degree> 90% of maximum) in the TSV group than in the V group. All hub taxa in the TSV group were bacteria, predominantly affiliated with the phyla Proteobacteria, Actinobacteriota, Cyanobacteria, Bdellovibrionota. Key genera included *Candidatus_Megaira*, *Cupriavidus*, *Pseudomonas*, *Amycolatopsis*, *Sphaerotilus*, *Phreatobacter*, *Ancylobacter*, and 7 unclassified genera. In contrast, hub taxa in the V group comprised both bacteria and eukaryotes. The bacterial hubs (*Phreatobacter*, SWB02, and *Sphingorhabdus*) belonged exclusively to Proteobacteria. Eukaryotic hubs consisted of the Choanoflagellate *Monosiga* and one unclassified fungus (**Figure 5A**).

### Enrichment of algicidal epiphytic bacteria

**Figure 6:**
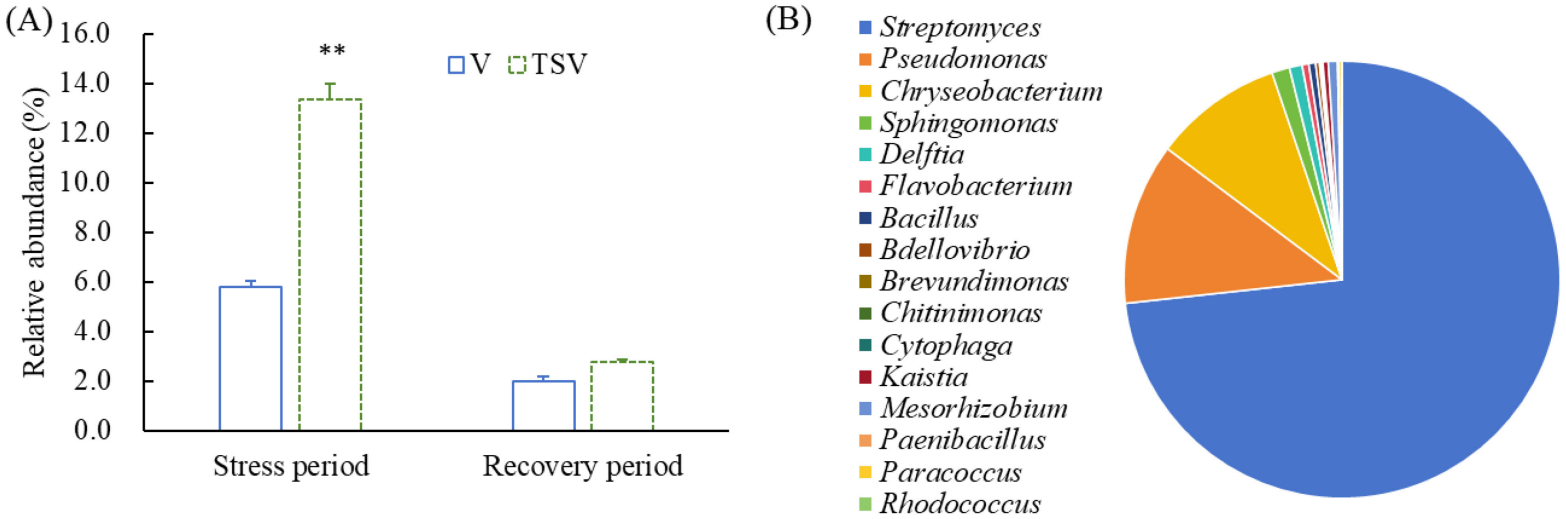
Total relative abundance of potential epiphytic algicidal bacteria in the V and TSV groups at the end of stress and recovery periods **(A)** and the predominant enriched genera in the TSV group at the end of stress period **(B)**. Data are means ± standard deviation analyzed from three parallel samples. ** indicates significant differences between two groups (*p* < 0.05).

We further screened 22 genera of bacteria known to be algicidal from the epiphytic microbiomes of *V. natans* (**Supplementary Table S2**). The relative abundance of the algicidal bacteria reached up to 13.4% in the TSV group, significantly higher than that in the V group (5.8%, *p* < 0.01) at the end of the stress period. However, this disparity between the TSV group and V group diminished during the recovery period (**Figure 6A**). The enriched algicidal bacteria in the TSV group predominantly belonged to the phyla Actinobacteriota, Proteobacteria, Firmicutes, Bacteroidota, and Bdellovibrionota. The first dominant algacidal bacteria was *Streptomyces* belonging to Actinobacteriota, accounting for 73%. The genera *Pseudomonas* and *Chryseobacterium* were second dominant, accounting for 12% and 10% respectively (**Figure 6B**).

The random forest model screened out top 10 metabolites that were significantly correlated with the algicidal bacterial community (*p* < 0.05), from 117 major differential metabolites in the treatment and control groups during the stress period. They are catechin 7-O-apiofuranoside, 2-(4-Methyl-3-cyclohexen-1-yl)-2-propanyl 6-O-(6-deoxy-L-mannopyranosyl) -D-glucopyranoside, jasmonic acid, clothianidin, fungichromin, n-(1,1-dioxotetrahydro-1H-1lambda-6-–thiophen-3-yl)-5-(2-pyridinyl)-2-thiophenecarboxamide, {(2R,4S,5R)-5-[1-Methyl-3-(2-naphthyl)-1H-pyrazol-5-yl]-1-azabicyclo[2.2.2]oct-2-yl}methyl [3-(trifluoromethyl)phenyl] carbamate, n-Acetyl-L-carnosine, propiomazine (**Supplementary Figure S3**). We also found that the relative abundance of metabolites (2R,3R)-3-Methylglutamyl-5-semialdehyde-N6-lysine, 1H-Indole-3-carboxaldehyde, 3-[3-(beta-D-Glucopyranosyloxy)- 2-hydroxyphenyl] propanoic acid and 2,5-di-tert-Butylhydroquinone in the TSV group was higher than that in the V group during the stress period, but not during the recovery period (*p* < 0.05, **Supplementary Figure S4**).

## Discussion

### Restructuring of epiphytic microbial community

While previous research has established that *Microcystis* can influence epiphytic bacterial communities on submerged macrophytes (Jiang et al., 2019; Li et al., 2020; Gao et al., 2022), temporal dynamics and the response of the entire microbial communities-including both prokaryotic and eukaryotic components- remain less understood. This study provides, to our knowledge, the first time-resolved evidence that the interaction with microcystin-producing *Microcystis* altered the structure and diversity of the entire epiphytic microbial community on the phyllosphere of the submerged macrophyte *V. natans*, with distinct responses observed between prokaryotic and eukaryotic microbial components. It has previously been reported that *V. natans* can reconstruct their epiphytic microbiome as a “cry for help” mechanism in response to acute ammonium stress (Hu et al., 2023). These findings suggest that aquatic plants may also utilize the “cry for help” strategy to defend against biotic and abiotic environmental stressors like terrestrial plants (Hu et al., 2018; Jiang et al., 2025; Zeng et al., 2025).

Taxon-specific responses of epiphytic microbiomes have been observed to differ between prokaryotic and eukaryotic communities across various life stages of aquatic plants and in response to diverse environmental changes, including warming, nutrient enrichment, pollutants, and hydrodynamic disturbances (Chen et al., 2022; Peng et al., 2024; Xia et al., 2020; Yang et al., 2025; Zhan et al., 2021). However, this is the first study to reveal that the diversity and community structure of epiphytic bacteria, algae, and other eukaryotes (mainly fungi and protozoa) on submerged macrophytes exhibit contrasting response dynamics to the emergence of microcystin-producing *Microcystis*. It is not only closely linked to their sensitivity to stress from *Microcystis* and microcystins, but is also shaped by the roles they played in facilitating plant stress resilience (Zeng et al., 2025).

The increased diversity of epiphytic bacteria and algae, coupled with reduced diversity of other epiphytic eukaryotes (**Figures 4A, B, C**), coincided with the complete disappearance of *Microcystis* cells, maximum extracellular microcystins and adverse effects on the growth and metabolism of *V. natans* at the end of the stress period in the TSV group (**Figures 2**, **3**). The decomposition process of *Microcystis* cells releases a substantial amount of inorganic and organic matter, including secondary metabolites such as microcystins (Peng et al., 2024). This provides a diverse array of nutritional resources for epiphytic bacteria and algae (Cui et al., 2025; Liu et al., 2024). Concurrently, the diverse planktonic bacteria surrounding the *Microcystis* cells may have chances to colonize the phyllosphere of submerged macrophytes (Gong and Xin, 2021; Gao et al., 2022; He et al., 2023; Tsai et al., 2025). These factors could collectively account for the significant increase in bacterial and algal community diversity on leaves by the end of the stress period. In contrast, other epiphytic eukaryotes-including fungi and protozoa-likely experience a marked reduction in diversity. This decline may be attributed to the toxic effects of microcystins released in large quantities into the aquatic environment (Mehinto et al., 2021), combined with alterations in the interaction network including cross-feeding, parasitism, symbiosis, and predatism among phyllosphere eukaryotes and bacteria (Manirakiza et al., 2022; Shi et al., 2025). During the subsequent recovery phase of the TSV group, the diversity of the epiphytic bacterial and eukaryotic community diversity recovered to a level close to that of the control group. This shift occurred alongside a rapid decrease in extracellular microcystin concentrations and the restoration of plant growth and metabolic activity. These findings indicate that as the *Microcystis* -induced stress subsided, the plant-phyllosphere microbiome symbiont exhibited considerable resilience, which likely supports more stable plant colonization in degraded eutrophic waterbodies (Hu et al., 2023).

At both the phylum and genus levels, the structural shifts in the entire epiphytic microbial community were significantly more pronounced in the TSV treatment group than in the control (**Supplementary Figure S1**). The ambient environmental changes induced by *Microcystis* stress likely constitute a key deterministic process driving the reassembly of the epiphytic microbiota, a conclusion further supported by community assembly mechanism analysis (**Supplementary Figure S2**). All hub taxa of co-occurrence network of epiphytic microbiomes in the TSV group is bacteria, which confirmed the important roles of epiphytic bacteria during the inhibition of host plant on *Microcystis*. Proteobacteria was the most dominant phylum of the epiphytic bacteria in the study, which is consistent with other survey results (Schlechter et al., 2019; Zhen et al., 2020b; Yu et al., 2022; Wang et al., 2024). However, the relative abundance of Actinobacteria in the TSV group increased significantly during both the stress and recovery stages. There are reports of some algicidal bacteria belonging to Actinobacteria, such as the genera *Rhodococcus* and *Streptomyces* (Morón-López et al., 2024). Interestingly, the relative abundance of *Streptomyces* in the TSV group significantly increased at the end of stress period, whereas it decreased markedly during the recovery period. Its primary mechanisms involved in algicidal impact have been shown to be mycelia-mediated cell-to-cell contact, which facilitates the formation of flocs as well as the secretion of active algicidal substances that lead to *Microcystis* cell death (Yu et al., 2019; Kong et al., 2020; Zeng et al., 2021). A higher abundance of *Reyranella* belonging to the phylum Proteobacteria was also observed during the stress and recovery period in the TSV group compared to the V group. *Reyranella* exhibits denitrification capabilities and an enhanced ability to degrade chemical oxygen demand (Duan et al., 2023; Zhang et al., 2025), which is likely involved in the degradation of organic carbon and nitrogen released from lysed *Microcystis* cells.

While submerged plants have been often demonstrated to be negatively impacted by epiphytic algae due to competition for limited light and nutrients (Song et al., 2017; Wijewardene et al., 2022), our experimental results revealed a distinct pattern. The structure of the epiphytic eukaryotic algal community (primarily diatoms) remained stable throughout the experiment, and its contribution to the microbial co-occurrence network was consistently low (< 10% in both groups, **Supplementary Table S1**). This suggests that eukaryotic algae were less responsive to *Microcystis* stress than the other epiphytic microbial community under the present conditions. Borrego-Ramos et al. (2019) observed higher diatom richness on dead macrophyte stems compared to living tissues. Eukaryotic algae in our study exhibited the lowest diversity among the three major epiphytic microbial groups. This low diversity and stable structure may correlate with reduced competitive pressure on the host plant for phyllosphere resources, thereby minimizing adverse impacts on *V. natans* (Jones et al., 2002).

The limited response of eukaryotic algae could be linked to top-down regulatory pressure from eukaryotic parasitoids. Notably, we observed a marked increase in the relative abundance of the genus *Paraphelidium* in the TSV group at the end of the stress (**Supplementary Figure S1**). *Paraphelidium* belongs to Aphelidea, a group of intracellular parasitoids known to infect eukaryotic algae such as diatoms and increase host mortality by up to 70%, thereby preventing excessive algal biofilm formation on submerged surfaces (Karpov et al., 2014; Traver-Azuara et al., 2025). We therefore hypothesize that Aphelidea may act as a key regulator of biofilm stability by suppressing the overgrowth of epiphytic algae, which in turn helps maintain colonization space for epiphytic bacteria. Nevertheless, this regulatory role requires further experimental validation.

### Recruitment of algicidal bacteria

Within the epiphytic bacterial community identified in this study, 22 bacterial genera previously reported to possess algicidal potential was detected (Deng et al., 2010; Yang et al., 2020; Coyne et al., 2022; Morón-López et al., 2024). Notably, the total relative abundance and diversity of these algicidal bacteria significantly increased during the stress period. During the recovery phase, their relative abundance decreased back to levels comparable with the control. This significant dynamic response, combined with our preliminary experiments and the detection of algicidal bacteria (Wei et al., 2024), leads us to propose that submerged macrophytes may recruit algicidal bacteria to enhance the suppression capability on *Microcystis*. Building upon the traditional perspective that submerged macrophytes directly suppress *Microcystis* via the release of allelochemicals (Gao et al., 2017; Gross et al., 2007; Hilt and Gross, 2008; Nakai et al., 2012), this study proposes an indirect inhibitory mechanism mediated by the recruitment of algicidal bacteria. Consistent with the key roles of epiphytic microbiota in regulating biological processes of terrestrial plants, such as nutrient acquisition and resistance to abiotic and biotic stresses (Gong and Xin, 2021), the epiphytic microbiota of submerged macrophytes also plays a non-negligible role in mediating host plants’ inhibitory effects against *Microcystis*.

It is well-established that terrestrial plants employ a “cry for help” strategy by exuding various primary and secondary metabolites. These compounds recruit beneficial environmental microbes, which in turn enhance the host plant’s stress tolerance and growth (Rizaludin et al., 2021; Rolli et al., 2021). Non-targeted metabolomics analysis exhibited at least 117 different metabolites and 16 differential metabolic pathways of *V. natans* in the TSV group compared to those in the V group at the end of the stress period. Among the 16 significantly perturbed metabolic pathways, glutathione metabolism and ABC transporter pathways were extensively documented in relation to plant detoxification and stress resistance (Dorion et al., 2021; Zhou et al., 2024). We speculate that metabolic diversification within the plant kingdom may provide a basis for communication and recognition that enables the sculpting of microbiota tailored to the needs of the host (Huang et al., 2019).

Jasmonic acid, identified as one of the key metabolites strongly associated with the epiphytic algicidal bacteria (**Supplementary Figure S3**), is a pivotal phytohormone. Beyond its well-established roles in regulating plant growth and development, jasmonic acid is a central mediator of plant defense signaling and plant-microbe interactions (Fresno and Munné-Bosch, 2021). A recent study has documented a significant positive correlation between jasmonic acid and the recruitment of beneficial drought-enriched bacteria that enhance plant fitness (Xiang et al., 2025). More specifically, jasmonic acid has been shown to directly regulate the growth, development, and specialized metabolism of *Streptomyces* (van der Meij et al., 2023), a genus that was notably enriched on the phyllosphere of *V. natans* during the stress phase in our experiment. This suggests that jasmonic acid likely functions as a crucial signaling molecule, coordinating the active recruitment and physiological activation of specific beneficial epiphytic bacteria under stress. However, the precise mechanisms by which plant-derived metabolites shape the structure and function of epiphytic microbial communities remain an important area for future investigation (Xu et al., 2022).

The screened algicidal bacteria mainly belonged to phyla phyla Proteobacteria, Firmicutes, Bacteroidota and Actinobacteriota. It has been reviewed that a large number of algicidal bacteria belong mainly to class Gammaproteobacteria (47%) and class Bacilli (31%) (phylum Firmicutes) (Morón-López et al., 2024). The dominant genera *Streptomyces*, *Pseudomonas* and *Chryseobacterium* have been reported to exert algicidal effects through direct and indirect modes (Guo et al., 2015; Yu et al., 2019; Zhang et al., 2019; Kong et al., 2020; Zeng et al., 2021). Some screened metabolites with the highest abundance at the end of the stress period in the TSV group, such as 1H-Indole-3-carboxaldehyde, have been identified from algicidal bacteria as algicidal substances (Kong et al., 2022). The algicidal modes and mechanisms of epiphytic bacteria from submerged macrophytes still need further studies on the basis of the isolation of algicidal bacteria.

We also found several algicidal bacteria had capability to degrade MCs, including *Pseudomonas*, *Sphingomonas*, and *Stenotrophomonas* belonging to Phylum Proteobacteria, *Bacillus* belonging to Phylum Firmicutes, *Chryseobacterium* belonging to phylum Bacteroidota, *Rhodococcus* belonging to phylum Actinobacteriota, *Streptomyces* belonging to phylum Actinobacteriota (Dziga et al., 2013; Kormas and Lymperopoulou, 2013; Zhang et al., 2016; Kumar et al., 2018). This suggests that epiphytic bacteria may play a role in the degradation of microcystins, although further experimental evidence is required to confirm their involvement.

**Figure 7:**
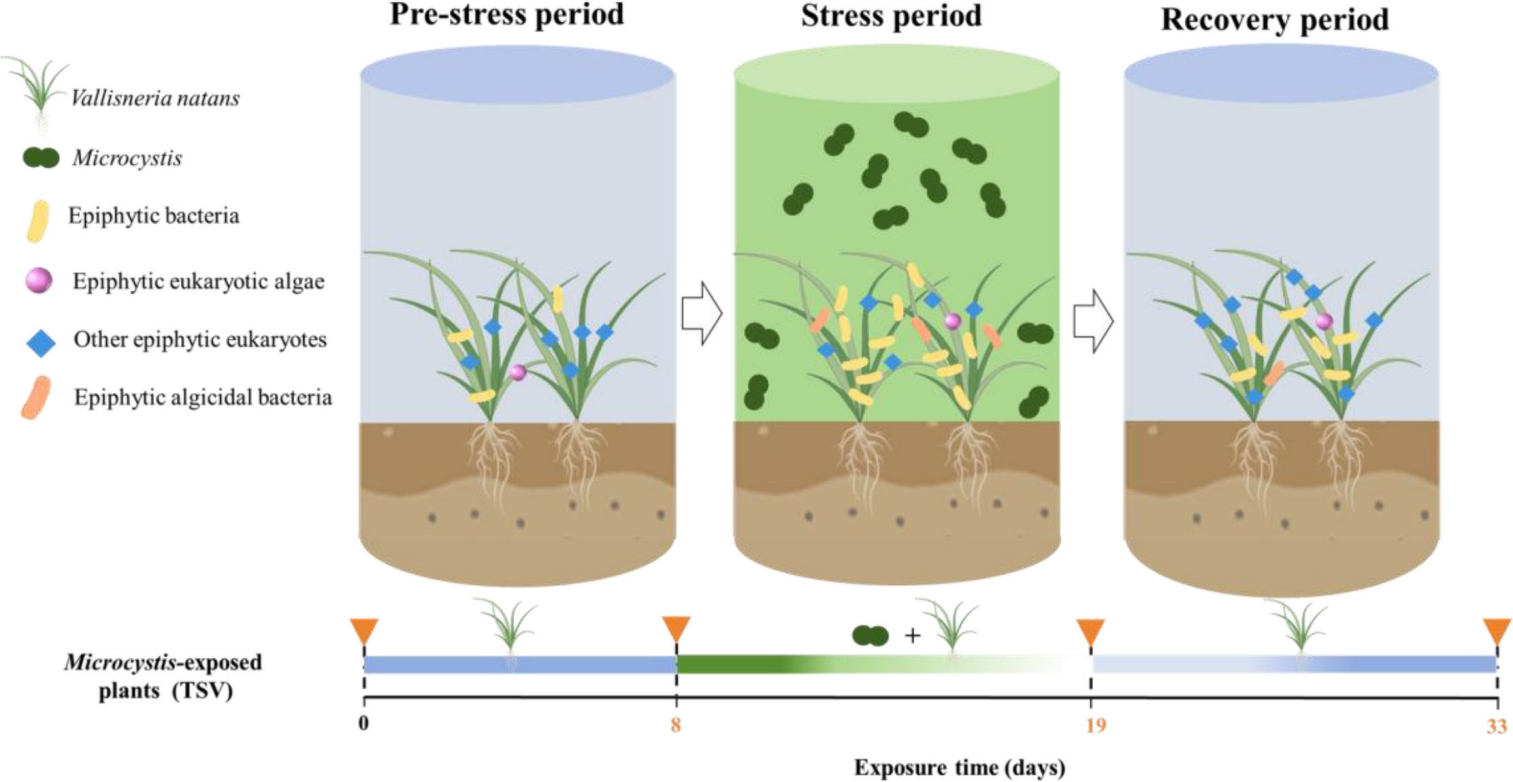
Schematic diagram of the restructuring of the epiphytic microbiome and recruitment of algicidal bacteria by *V. natans* for the suppression of *Microcystis*.

Based on our experimental findings, we propose that the submerged macrophyte *V. natans* restructures its epiphytic microbial communities and recruits algicidal bacteria to suppress *Microcystis*. Specifically, during the stress period—characterized by the gradual disappearance of *Microcystis* cells—the alpha-diversity of epiphytic bacteria increased significantly, while that of epiphytic eukaryotes (including fungi and protozoa) decreased markedly. In the subsequent recovery phase, the alpha-diversity of the epiphytic microbial community returned to a level comparable to that of the control plants. Throughout these phases, the structure and diversity of epiphytic microbiomes (with the exception of eukaryotic algae) shifted dynamically. Notably, epiphytic bacteria contributed most substantially to the interactions among the three major epiphytic microbial groups in response to *Microcystis* stress. The enrichment of algicidal bacteria on the phyllosphere during the stress phase likely played a key role in the successful suppression of *Microcystis* by *V. natans* (**Figure 7**).

## Conclusions

This study represents the first comprehensive demonstration of epiphytic microbiome restructuring in response to *Microcystis*-induced stress and subsequent recovery of the plant. Notably, algicidal bacteria were exclusively enriched at the stress-phase, indicating their potential involvement in host-mediated inhibition on *Microcystis*. Through a time-series analysis, we provide evidence that the presence of the stressor triggers a “cry for help” response between submerged macrophytes and their epiphytic microorganisms, thereby offering novel insights into the dynamic community-level mechanisms by which microorganisms contribute to host plants’ inhibition on *Microcystis*.

## Data Availability

The datasets presented in this study can be found in online repositories. The names of the repository/repositories and accession number(s) can be found in the article/**Supplementary Material**.

## References

[B1] Berendsen R. L. Vismans G. Yu K. Song Y. De Jonge R. Burgman W. P. . (2018). Disease-induced assemblage of a plant-beneficial bacterial consortium. ISME J. 12, 1496–1507. doi: 10.1038/s41396-018-0093-1, PMID: 29520025 PMC5956071

[B2] Borrego-Ramos M. Olenici A. Blanco S. (2019). Are dead stems suitable substrata for diatom-based monitoring in Mediterranean shallow ponds? Fundam. Appl. Limnol./Arch. für Hydrobiol. 192, 215–224. doi: 10.1127/fal/2019/1163

[B3] Chen H. Zhang S. Lv X. Guo S. Ma Y. Han B. (2022). Interactions between suspended sediments and submerged macrophytes-epiphytic biofilms under water flow in shallow lakes. Water Res. 222, 118911. doi: 10.1016/j.watres.2022.118911, PMID: 35932704

[B4] Coyne K. J. Wang Y. Johnson G. (2022). Algicidal bacteria: a review of current knowledge and applications to control harmful algal blooms. Front. Microbiol. 13, 871177. doi: 10.3389/fmicb.2022.871177, PMID: 35464927 PMC9022068

[B5] Cui D. He H. Zhang Z. Liu F. Gui Y. Guo Z. . (2025). Coupling of algae, algal organic matter, and nutrient biogeochemical cycling in eutrophic waters. Rev. Environ. Sci. Biotechnol. 24, 451–475. doi: 10.1007/S11157-025-09731-9/TABLES/2

[B6] Danso Ofori A. Su W. Zheng T. Datsomor O. Titriku J. K. Xiang X. . (2024). Roles of phyllosphere microbes in rice health and productivity. Plants. 13, 3268. doi: 10.3390/plants13233268, PMID: 39683062 PMC11644232

[B7] Deng H. Li Q. Li M. Sun L. Li B. Wang Y. . (2024). Epiphytic microorganisms of submerged macrophytes effectively contribute to nitrogen removal. Environ. Res. 242, 117754. doi: 10.1016/j.envres.2023.117754, PMID: 38016497

[B8] Deng J. Tao Y. Li D. Dong J. (2010). Advances in research of algicidal bacteria. Chin. J. Appl. Environ. Biol. 2009, 895–900. doi: 10.3724/SP.J.1145.2009.00895

[B9] Dorion S. Ouellet J. C. Rivoal J. (2021). Glutathione metabolism in plants under stress: beyond reactive oxygen species detoxification. Metabolites 11, 641. doi: 10.3390/METABO11090641, PMID: 34564457 PMC8464934

[B10] Duan M. Wang L. Song X. Zhang X. Wang Z. Lei J. . (2023). Assessment of the rhizosphere fungi and bacteria recruited by sugarcane during smut invasion. Braz. J. Microbiol. 54, 385–395. doi: 10.1007/s42770-022-00871-6, PMID: 36371518 PMC9944363

[B11] Dziga D. Wasylewski M. Wladyka B. Nybom S. Meriluoto J. (2013). Microbial degradation of microcystins. Chem. Res. Toxicol. 26, 841–852. doi: 10.1021/tx4000045, PMID: 23621464

[B12] Fresno D. H. Munné-Bosch S. (2021). Differential tissue-specific jasmonic acid, salicylic acid, and abscisic acid dynamics in sweet cherry development and their implications in fruit-microbe interactions. Front. Plant Sci. 12, 640601. doi: 10.3389/FPLS.2021.640601, PMID: 33603766 PMC7884454

[B13] Gao Y. N. Dong J. Fu Q. Q. Wang Y. P. Chen C. Li J. H. . (2017). Allelopathic effects of submerged macrophytes on phytoplankton. Allelopathy J. 40, 1–22. doi: 10.26651/2017-40-1-1062

[B14] Gao Y. Yang H. Gao X. Li M. Zhang M. Dong J. . (2022). Ecological damage of submerged macrophyte *Myriophyllum* sp*icatum* by cell extracts from microcystin (MC)- and non-MC-producing cyanobacteria, *Microcystis*. J. Oceanol. Limnol 40, 1732–1749. doi: 10.1007/s00343-022-1449-y

[B15] Geng N. Xia Y. Lu D. Bai Y. Zhao Y. Wang H. . (2022). The bacterial community structure in epiphytic biofilm on submerged macrophyte *Potamogetom crispus* L. and its contribution to heavy metal accumulation in an urban industrial area in Hangzhou. J. Hazard. Mater 430, 128455. doi: 10.1016/j.jhazmat.2022.128455, PMID: 35739657

[B16] Gong T. Xin X. F. (2021). Phyllosphere microbiota: community dynamics and its interaction with plant hosts. J. Integr. Plant Biol. 63, 297–304. doi: 10.1111/jipb.13060, PMID: 33369158

[B17] Gross E. M. Hilt S. Lombardo P. Mulderij G. (2007). Searching for allelopathic effects of submerged macrophytes on phytoplankton—state of the art and open questions. Hydrobiologia 584, 77–88. doi: 10.1007/s10750-007-0591-z

[B18] Guo X. Liu X. Pan J. Yang H. (2015). Synergistic algicidal effect and mechanism of two diketopiperazines produced by *Chryseobacterium* sp. strain GLY-1106 on the harmful bloom-forming *Microcystis aeruginosa*. Sci. Rep. 5, 1–12. doi: 10.1038/srep14720, PMID: 26423356 PMC4589682

[B19] He R. Hu S. Li Q. Zhao D. Wu Q. L. Zeng J. (2023). Greater transmission capacities and small-world characteristics of bacterial communities in the above- than those in the below- ground niches of a typical submerged macrophyte, *Vallisneria natans*. Sci. Total Environ. 903, 166229. doi: 10.1016/j.scitotenv.2023.166229, PMID: 37586539

[B20] Hilt S. Gross E. M. (2008). Can allelopathically active submerged macrophytes stabilise clear-water states in shallow lakes? Basic Appl. Ecol. 9, 422–432. doi: 10.1016/j.baae.2007.04.003

[B21] Hu S. He R. He X. Zeng J. Zhao D. (2023). Niche-specific restructuring of bacterial communities associated with submerged macrophyte under ammonium stress. Appl. Environ. Microbiol. 89, e00717–23. doi: 10.1128/aem.00717-23, PMID: 37404156 PMC10370296

[B22] Hu L. Robert C. A. M. Cadot S. Zhang X. Ye M. Li B. . (2018). Root exudate metabolites drive plant-soil feedbacks on growth and defense by shaping the rhizosphere microbiota. Nat. Commun. 9, 2738. doi: 10.1038/s41467-018-05122-7, PMID: 30013066 PMC6048113

[B23] Huang A. C. Jiang T. Liu Y. X. Bai Y. C. Reed J. Qu B. . (2019). A specialized metabolic network selectively modulates *Arabidopsis* root microbiota. Science 364, eaau6389. doi: 10.1126/science.aau6389, PMID: 31073042

[B24] Jeong S. Yang D. Joo S. Park S. (2021). Allelopathic inhibition effects of *Myriophyllum* sp*icatum on* growths of bloom-forming cyanobacteria and other phytoplankton species in coexistence experiments. J. Plant Biol. 64, 501–510. doi: 10.1007/s12374-021-09322-5

[B25] Jiang X. Wang M. Yang S. He D. Fang F. Yang L. (2024). The response of structure and nitrogen removal function of the biofilm on submerged macrophytes to high ammonium in constructed wetlands. J. Environ. Sci. 142, 129–141. doi: 10.1016/j.jes.2023.07.004, PMID: 38527879

[B26] Jiang H. Xu X. Lv L. Huang X. Ahmed T. Tian Y. . (2025). Host metabolic alterations mediate phyllosphere microbes defense upon xanthomonas oryzae pvoryzae infection. J. Agric. Food Chem. 73, 249–259. doi: 10.1021/acs.jafc.4c09178, PMID: 39690815

[B27] Jiang M. Zhou Y. Wang N. Xu L. Zheng Z. Zhang J. (2019). Allelopathic effects of harmful algal extracts and exudates on bio films on leaves of *Vallisneria natans*. Sci. Total Environ. 655, 823–830. doi: 10.1016/j.scitotenv.2018.11.296, PMID: 30481709

[B28] Jones J. I. Young J. O. Eaton J. W. Moss B. (2002). The influence of nutrient loading,dissolved inorganic carbon and higher trophic levels on the interaction between submerged plants and periphyton. J. Ecol. 90, 12–24. doi: 10.1046/j.0022-0477.2001.00620.x

[B29] Karpov S. A. Mamkaeva M. A. Aleoshin V. V. Nassonova E. Lilje O. Gleason F. H. (2014). Morphology, phylogeny, and ecology of the *Aphelids* (Aphelidea, Opisthokonta) and proposal for the new superphylum *Opisthosporidia*. Front. Microbiol. 5, 112. doi: 10.3389/FMICB.2014.00112, PMID: 24734027 PMC3975115

[B30] Kong Y. Wang Q. Chen Y. Xu X. Zhu L. Yao H. . (2020). Anticyanobacterial process and action mechanism of *Streptomyces* sp. HJC-D1 on *Microcystis aeruginosa*. Environ. Prog. Sustain. Energy 39. doi: 10.1002/ep.13392

[B31] Kong Y. Wang Y. Miao L. Mo S. Li J. Zheng X. (2022). Recent advances in the research on the anticyanobacterial effects and biodegradation mechanisms of *Microcystis aeruginosa* with microorganisms. Microorganisms 10, 1136. doi: 10.3390/microorganisms10061136, PMID: 35744654 PMC9229865

[B32] Kormas K. A. Lymperopoulou D. S. (2013). Cyanobacterial toxin degrading bacteria: Who are they? BioMed. Res. Int. 2013, 463894. doi: 10.1155/2013/463894, PMID: 23841072 PMC3690202

[B33] Kumar P. Hegde K. Brar S. K. Cledon M. Kermanshahi-pour A. Roy-Lachapelle A. . (2018). Biodegradation of microcystin-LR using acclimatized bacteria isolated from different units of the drinking water treatment plant. Environ. pollut. 242, 407–416. doi: 10.1016/J.ENVPOL.2018.07.008, PMID: 30005254

[B34] Li Q. Gu P. Zhang H. Luo X. Zhang J. Zheng Z. (2020). Response of submerged macrophytes and leaf biofilms to the decline phase of *Microcystis aeruginosa*: antioxidant response, ultrastructure, microbial properties, and potential mechanism. Sci. Total Environ. 699, 134325. doi: 10.1016/j.scitotenv.2019.134325, PMID: 31678882

[B35] Li B. Li J. An G. Zhao C. Wang C. (2021). Long-term and strong suppression against Microcystis growth and microcystin-release by luteolin continuous-release microsphere: Optimal construction, characterization, effects and proteomic mechanisms. Water Res. 202, 117448. doi: 10.1016/j.watres.2021.117448, PMID: 34364065

[B36] Li X. Liu W. Ge Y. Shi R. Yin C. Liu J. . (2024). Response of *Ceratophyllum demersum* L. and its epiphytic biofilms to 6PPD and 6PPD-Q exposure: Based on metabolomics and microbial community analysis. J. Hazard. Mater. 480, 136420. doi: 10.1016/j.jhazmat.2024.136420, PMID: 39509872

[B37] Liu X. Sun T. Yang W. Li X. Ding J. Fu X. (2024). Meta-analysis to identify inhibition mechanisms for the effects of submerged plants on algae. J. Environ. Manage 355, 120480. doi: 10.1016/j.jenvman.2024.120480, PMID: 38430885

[B38] Lu J. Mu X. Zhang S. Song Y. Ma Y. Luo M. . (2023). Coupling of submerged macrophytes and epiphytic biofilms reduced methane emissions from wetlands: evidenced by an antibiotic inhibition experiment. Sci. Total Environ. 904, 166710. doi: 10.1016/j.scitotenv.2023.166710, PMID: 37652383

[B39] Lu Z. Sha J. Tian Y. Zhang X. Liu B. Wu Z. (2017). Polyphenolic allelochemical pyrogallic acid induces caspase-3(like)-dependent programmed cell death in the cyanobacterium *Microcystis aeruginosa*. Algal Res. 21, 148–155. doi: 10.1016/j.algal.2016.11.007

[B40] Manirakiza B. Zhang S. Addo F. G. Isabwe A. Nsabimana A. (2022). Exploring microbial diversity and ecological function of epiphytic and surface sediment biofilm communities in a shallow tropical lake. Sci. Total Environ. 808, 151821. doi: 10.1016/j.scitotenv.2021.151821, PMID: 34808175

[B41] Mehinto A. C. Smith J. Wenger E. Stanton B. Linville R. Brooks B. W. . (2021). Synthesis of ecotoxicological studies on cyanotoxins in freshwater habitats – evaluating the basis for developing thresholds protective of aquatic life in the United States. Sci. Total Environ. 795, 148864. doi: 10.1016/J.SCITOTENV.2021.148864, PMID: 34328929

[B42] Morón-López J. Serwecińska L. Balcerzak Ł. Glińska S. Mankiewicz-Boczek J. (2024). Algicidal bacteria against cyanobacteria: practical knowledge from laboratory to application. Crit. Rev. Environ. Sci. Technol. 54(3), 239–266. doi: 10.1080/10643389.2023.2232257

[B43] Nakai S. Zou G. Okuda T. Nishijima W. Hosomi M. Okada M. (2012). Polyphenols and fatty acids responsible for anti-cyanobacterial allelopathic effects of submerged macrophyte *Myriophyllum* sp*icatum*. Water Sci. Technol. 66, 993–999. doi: 10.2166/wst.2012.272, PMID: 22797226

[B44] Ni L. Li X. Xu C. Li Y. Wu H. Du C. . (2021). Stress of artemisinin sustained-release granules on photosystem II, reactive oxygen species and metabolic activity of *Microcystis aeruginosa* cells. Bull. Environ. Contam. Toxicol. 107, 343–350. doi: 10.1007/s00128-021-03327-z, PMID: 34251462

[B45] Ning D. Deng Y. Tiedje J. M. Zhou J. (2019). A general framework for quantitatively assessing ecological stochasticity. Proc. Natl. Acad. Sci. U.S.A. 116, 16892–16898. doi: 10.1073/PNAS.1904623116, PMID: 31391302 PMC6708315

[B46] Ohore O. E. Zhang S. Guo S. Addo F. G. Manirakiza B. Zhang W. (2021). Ciprofloxacin increased abundance of antibiotic resistance genes and shaped microbial community in epiphytic biofilm on *Vallisneria* sp*iralis in* mesocosmic wetland. Bioresour. Technol. 323, 124574. doi: 10.1016/j.biortech.2020.124574, PMID: 33412499

[B47] Peng X. Lin Q. Liu B. Huang S. Yan W. Zhang L. . (2022). Effect of submerged plant coverage on phytoplankton community dynamics and photosynthetic activity in *situ*. J. Environ. Manage 301, 113822. doi: 10.1016/j.jenvman.2021.113822, PMID: 34607135

[B48] Peng T. Tang Y. Cai D. Gu Y. Wei J. Zhang J. . (2024). Insights into the interaction mechanisms between microcystin-degrading bacteria and *Microcystis aeruginosa*. Water Res. 265, 122241. doi: 10.1016/j.watres.2024.122241, PMID: 39154396

[B49] Perreault R. Laforest-Lapointe I. (2022). Plant-microbe interactions in the phyllosphere: facing challenges of the anthropocene. ISME J. 16, 339–345. doi: 10.1038/s41396-021-01109-3, PMID: 34522008 PMC8776876

[B50] Qin Z. Zhao Z. Xia L. Ohore O. E. (2022). Unraveling the ecological mechanisms of bacterial succession in epiphytic biofilms on *Vallisneria natans* and *Hydrilla verticillata* during bioremediation of phenanthrene and pyrene polluted wetland. J. Environ. Manage 321, 115986. doi: 10.1016/j.jenvman.2022.115986, PMID: 35998537

[B51] Ren Y. Yu G. Shi C. Liu L. Guo Q. Han C. (2022). Majorbio cloud: a onestop, comprehensive bioinformatic platform for multiomics analyses. iMeta 1, e12. doi: 10.1002/imt2.12, PMID: 38868573 PMC10989754

[B52] Rizaludin M. S. Stopnisek N. Raaijmakers J. M. Garbeva P. (2021). The chemistry of stress: understanding the ‘cry for help’ of plant roots. Metabolites. 11, 357. doi: 10.3390/metabo11060357, PMID: 34199628 PMC8228326

[B53] Rolli E. Vergani L. Ghitti E. Patania G. Mapelli F. Borin S. (2021). ‘Cry-for-help’ in contaminated soil: a dialogue among plants and soil microbiome to survive in hostile conditions. Environ. Microbiol. 23 (10), 5690–5703. doi: 10.1111/1462-2920.15647, PMID: 34139059 PMC8596516

[B54] Scheffer M. Carpenter S. Foley J. A. Folke C. Walker B. (2001). Catastrophic shifts in ecosystems. Nature 413, 591–596. doi: 10.1038/35098000, PMID: 11595939

[B55] Schlechter R. O. Miebach M. Remus-Emsermann M. N. P. (2019). Driving factors of epiphytic bacterial communities: A review. J. Adv. Res. 19, 57–65. doi: 10.1016/j.jare.2019.03.003, PMID: 31341670 PMC6630024

[B56] Shi R. Lian Y. Zeb A. Liu J. Yu M. Wang Q. . (2025). Foliar exposure to microplastics disrupts lettuce metabolism and negatively interferes with symbiotic microbial communities. Plant Physiol. Biochem. 223, 109823. doi: 10.1016/J.PLAPHY.2025.109823, PMID: 40147322

[B57] Sohrabi R. Paasch B. C. Liber J. A. Yang He S. (2023). Phyllosphere microbiome. Annu. Rev. Plant Biol. 74, 539–568. doi: 10.1146/annurev-arplant-102820 36854478

[B58] Song Y. Z. Wang J. Q. Gao Y. X . (2017). Effects of epiphytic algae on biomass and physiology of *Myriophyllum spicatum* L. with the increase of nitrogen and phosphorus availability in the water body. Environ. Sci. Pollut. Res. 24 (10), 9548–9555. doi: 10.1007/s113, PMID: 28243961

[B59] Sun S. Jian Z. Rao Q. Chen J. F. Zhong M. Wang Y. H. . (2024). Diversity of carbon cycle-linked phyllosphere microorganisms: a key driver of CO_2_ flux in macrophyte-dominated aquatic systems. Water Biol. Secur. 3, 100289. doi: 10.1016/j.watbs.2024.100289

[B60] Švanys A. Paškauskas R. Hilt S. (2014). Effects of the allelopathically active macrophyte *Myriophyllum* sp*icatum* on a natural phytoplankton community: a mesocosm study. Hydrobiologia 737, 57–66. doi: 10.1007/s10750-013-1782-4

[B61] Traver-Azuara J. Giner C. R. García-Comas C. Sánchez-Zurano A. Ciardi M. Acién G. . (2025). Complex interplay between the microalgae and their microbiome in production raceways. Bioresour Technol. 432, 132650. doi: 10.1016/j.biortech.2025.132650, PMID: 40349792

[B62] Tsai H. H. Tang Y. Jiang L. Xu X. Dénervaud Tendon V. Pang J. . (2025). Localized glutamine leakage drives the spatial structure of root microbial colonization. Science 390, eadu4235. doi: 10.1126/SCIENCE.ADU4235, PMID: 41037624

[B63] van der Meij A. Elsayed S. S. Du C. Willemse J. Wood T. M. Martin N. I. . (2023). The plant stress hormone jasmonic acid evokes defensive responses in streptomycetes. Appl. Environ. Microbiol. 89, e01239–23. doi: 10.1128/AEM.01239-23, PMID: 37902333 PMC10686085

[B64] Wang X. Liu Y. Qing C. Zeng J. Dong J. Xia P. (2024). Analysis of diversity and function of epiphytic bacterial communities associated with macrophytes using a metagenomic approach. Microb. Ecol. 87, 37. doi: 10.1007/s00248-024-02346-7, PMID: 38286834 PMC10824801

[B65] Wei Y. Gao Y. Li L. Zhao J. Zeng D. Zhang L. . (2024). Inhibition of *Microcystis aeruginosa* by submerged macrophytes and epiphytic biofilms. Chin. J. Appl. Ecoclogy 35, 3377–3385. doi: 10.13287/j.1001-9332.202412.003, PMID: 40383888

[B66] Wijewardene L. Wu N. Fohrer N. Riis T. (2022). Epiphytic biofilms in freshwater and interactions with macrophytes: Current understanding and future directions. Aquat. Bot. 176, 103467. doi: 10.1016/j.aquabot.2021.103467

[B67] Xia P. Yan D. Sun R. Song X. Lin T. Yi Y. (2020). Community composition and correlations between bacteria and algae within epiphytic biofilms on submerged macrophytes in a plateau lake, southwest China. Sci. Total Environ. 727, 138398. doi: 10.1016/j.scitotenv.2020.138398, PMID: 32335447

[B68] Xiang Q. Yang K. Cui L. Sun A. Q. Lu C. Y. Gao J. Q. . (2025). Global exploration of drought-tolerant bacteria in the wheat rhizosphere reveals microbiota shifts and functional taxa enhancing plant resilience. Nat. Food 6, 1054–1067. doi: 10.1038/s43016-025-01248-2, PMID: 41073839

[B69] Xu N. Zhao Q. Zhang Z. Zhang Q. Wang Y. Qin G. . (2022). Phyllosphere microorganisms: Sources, drivers, and their interactions with plant hosts. J. Agric. Food Chem. 70, 4860–4870. doi: 10.1021/acs.jafc.2c01113, PMID: 35435673

[B70] Yang H. Cao Y. Zhang W. Pacheco J. P. Liu T. Zheng Y. . (2025). Prokaryotic and eukaryotic periphyton responses to warming, nutrient enrichment and small omnivorous fish: a shallow lake mesocosms experiment. Environ. Res. 269, 120942. doi: 10.1016/J.ENVRES.2025.120942, PMID: 39870344

[B71] Yang C. Hou X. Wu D. Chang W. Zhang X. Dai X. . (2020). The characteristics and algicidal mechanisms of cyanobactericidal bacteria, a review. World J. Microbiol. Biotechnol. 36, 1–10. doi: 10.1007/s11274-020-02965-5, PMID: 33241509

[B72] Yang C. Shen X. Shi X. Cui Z. Nan J. Lu H. . (2023). Impact of submerged macrophytes on growth and 2-MIB release risk of *Pseudanabaena* sp.: from field monitoring to cultural experiments. J. Hazard. Mater. 442, 130052. doi: 10.1016/j.jhazmat.2022.130052, PMID: 36182878

[B73] Yu W. Li J. H. Ma X. Lv T. Wang L. Li J. R. . (2022). Community structure and function of epiphytic bacteria attached to three submerged macrophytes. Sci. Total Environ. 835, 155546. doi: 10.1016/j.scitotenv.2022.155546, PMID: 35489510

[B74] Yu Y. Zeng Y. Li J. Yang C. Zhang X. Luo F. . (2019). An algicidal *Streptomyces amritsarensis* strain against *Microcystis aeruginosa* strongly inhibits microcystin synthesis simultaneously. Sci. Total Environ. 650, 34–43. doi: 10.1016/j.scitotenv.2018.08.433, PMID: 30195130

[B75] Zeng Q. Hu H. W. Ge A. H. Xiong C. Zhai C. C. Duan G. L. . (2025). Plant–microbiome interactions and their impacts on plant adaptation to climate change. J. Integr. Plant Biol. 00, 1–19. doi: 10.1111/jipb.13863, PMID: 39981843

[B76] Zeng Y. Wang J. Yang C. Y. Ding M. Hamilton P. B. Zhang X. . (2021). A *Streptomyces globisporus* strain kills *Microcystis aeruginosa* via cell-to-cell contact. Sci. Total Environ. 769, 144489. doi: 10.1016/j.scitotenv.2020.144489, PMID: 33465632

[B77] Zhan P. Liu Y. Wang H. C. Wang C. Xia M. Wang N. . (2021). Plant litter decomposition in wetlands is closely associated with phyllospheric fungi as revealed by microbial community dynamics and co-occurrence network. Sci. Total Environ. 753, 142194. doi: 10.1016/j.scitotenv.2020.142194, PMID: 33207455

[B78] Zhang B. H. Ding Z. G. Li H. Q. Mou X. Z. Zhang Y. Q. Yang J. Y. . (2016). Algicidal activity of *Streptomyces eurocidicus* JXJ-0089 metabolites and their effects on *Microcystis* physiology. Appl. Environ. Microbiol. 82, 5132–5143. doi: 10.1128/AEM.01198-16, PMID: 27316950 PMC4988176

[B79] Zhang R. X. Liu Y. Huang D. N. Zhang L. Ma X. G. Yu P. F. . (2025). Influence of influent load on nitrification/denitrification with MBBR for oil shale retorting wastewater treatment: Performance and microbial community structure. Water Air Soil. pollut. 236, 4. doi: 10.1007/s11270-024-07615-2

[B80] Zhang C. Massey I. Y. Liu Y. Huang F. Gao R. Ding M. . (2019). Identification and characterization of a novel indigenous algicidal bacterium *Chryseobacterium* species against *Microcystis aeruginosa*. J. Toxicol. Environ. Health-Part A 82, 845–853. doi: 10.1080/15287394.2019.1660466, PMID: 31462174

[B81] Zhen Z. Yan C. Zhao Y. (2020a). Influence of epiphytic bacteria on arsenic metabolism in *Hydrilla verticillata*. Environ. pollut. 261, 114232. doi: 10.1016/j.envpol.2020.114232, PMID: 32114122

[B82] Zhen Z. Yan C. Zhao Y. (2020b). Epiphytic bacterial community enhances arsenic uptake and reduction by *Myriophyllum verticillatum*. Environ. Sci. pollut. Res. 27, 44205–44217. doi: 10.1007/s11356-020-10274-5, PMID: 32757129

[B83] Zhou Y. Wang Y. Zhang D. Liang J. (2024). Endomembrane-biased dimerization of ABCG16 and ABCG25 transporters determines their substrate selectivity in ABA-regulated plant growth and stress responses. Mol. Plant 17, 478–495. doi: 10.1016/J.MOLP.2024.02.005, PMID: 38327051

[B84] Zhu J. Liu B. Wang J. Gao Y. Wu Z. (2010). Study on the mechanism of allelopathic influence on cyanobacteria and chlorophytes by submerged macrophyte (*Myriophyllum* sp*icatum*) and its secretion. Aquat. Toxicol. 98, 196–203. doi: 10.1016/j.aquatox.2010.02.011, PMID: 20451264

