# Supplementary material for: Restructuring of the epiphytic microbiome and recruitment of algicidal bacteria by Vallisneria natans for the suppression of Microcystis
Source: Front Plant Sci. 2026 Jan 14;16:1731742. doi: 10.3389/fpls.2025.1731742 (PMC12847314; doi:10.3389/fpls.2025.1731742)
Supplement: Supplementary file 1 [file DataSheet1.docx]

**Restructuring of the epiphytic microbiome and recruitment of algicidal bacteria by *Vallisneria natans* for the suppression of *Microcystis***

**Supplementary materials**

Yunni Gao^1,2,3*^, Ying Wei^1,2,3^, Dahai Zeng^1,2,3^, Jingxiao Zhang^1,2,3^, Jing Dong^1,2,3^, Xiaofei Gao^1,2,3^, Huatao Yuan^1,2,3^, Xuejun Li^1,2,3*^, Dongru Qiu^4^, Michele Burford^5^

1. College of Fisheries, Henan Normal University, Xinxiang, China;

2. Observation and Research Station on Water Ecosystem in Danjiangkou Reservoir of Henan Province，Nanyang , China;

3. The National Ecological Quality Comprehensive Monitoring Station (Hebi Station), Hebi , China.

4. Institute of Hydrobiology, Chinese Academy of Sciences, Wuhan , China

5. Australian Rivers Institute, Griffith University, 68 University Dr, Meadowbrook, Queensland 4131, Australia

*Corresponding Author：Yunni Gao, E-mail: gaoyn@htu.cn; Xuejun Li，E-mail: xjli@htu.cn


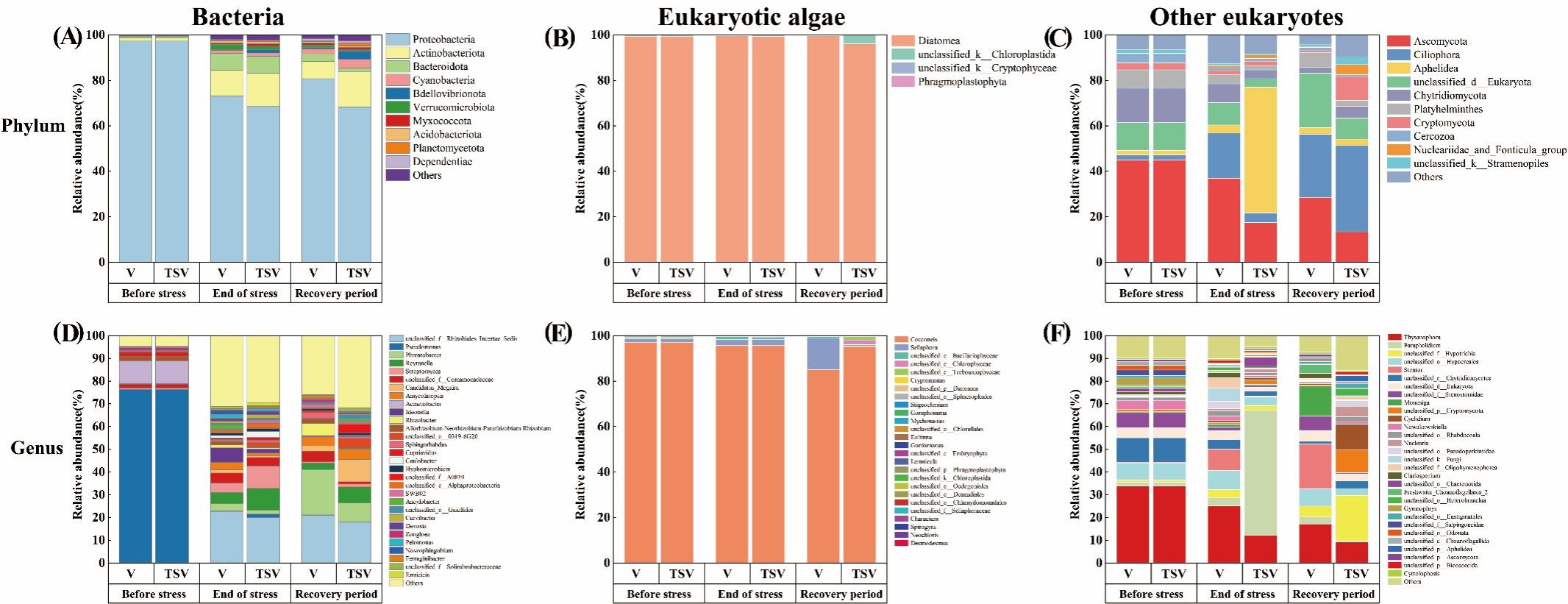


Figure S1 Community composition of epiphytic bacteria (A, D), eukaryotic algae (B, E) and other eukaryotes (C, F) at phylum (upper) and genus (lower) levels.


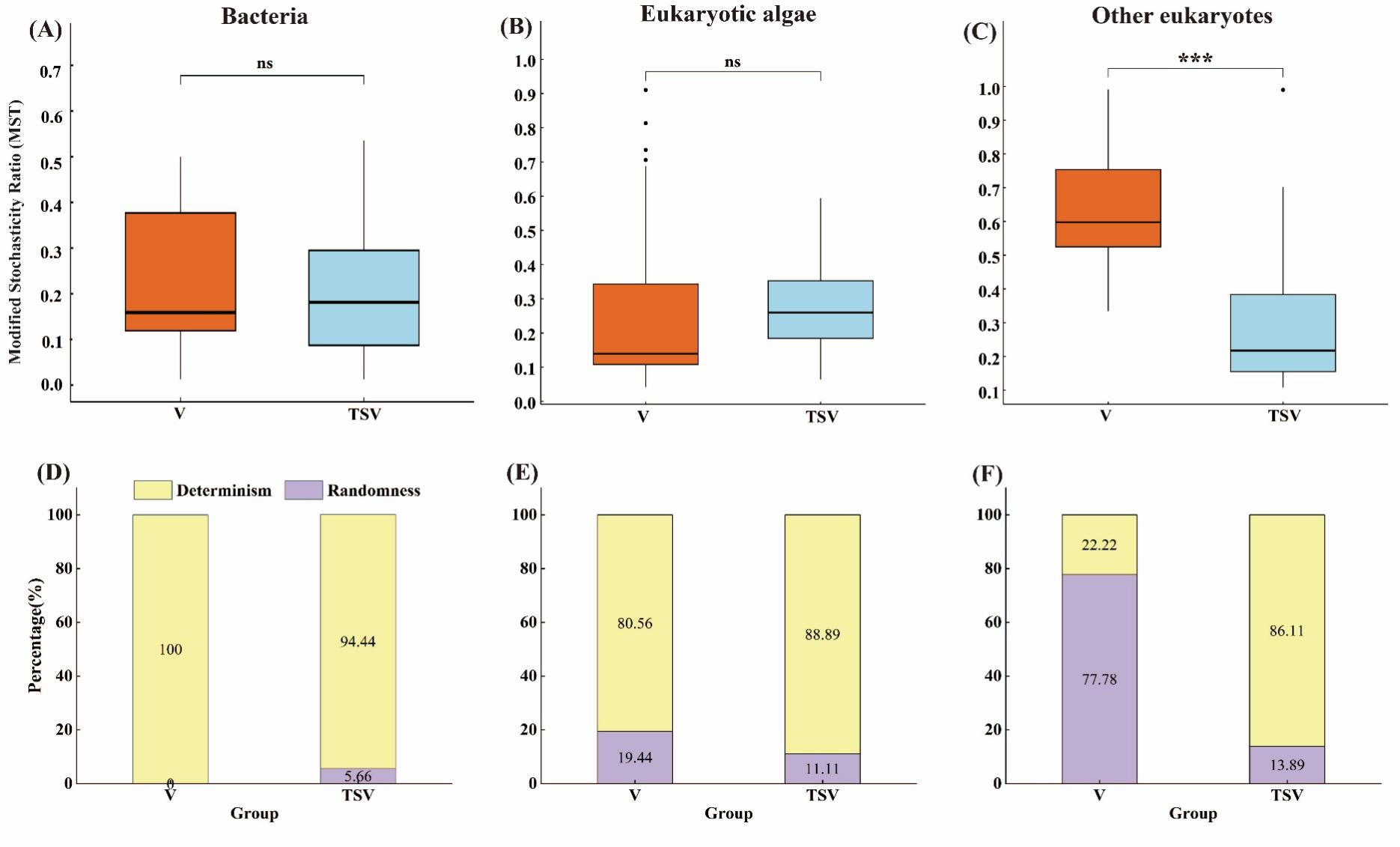


Figure S2 Assembly (A, B, C) and contribution patterns (D, E, F) of epiphytic bacteria, eukaryotic algae and the other eukaryotes.

Table S1 Key topological features of epiphytic microbial community networks.

| Network properties | V | TSV |
| --- | --- | --- |
| Number of nodes | 45 | 74 |
| Number of edges | 235 | 666 |
| Network density | 0.227 | 0.273 |
| Modularity | 0.413 | 0.43 |
| Bacteria (%) | 62.22 | 65.31 |
| Eukaryotic algae (%) | 8.89 | 8.16 |
| Other eukaryotes (%) | 28.89 | 26.53 |
| Proportion of positive edges (%) | 63.4 | 56.89 |
| Proportion of negative edges (%) | 36.6 | 43.11 |

Table S2 The screened epiphytic algicidal bacteria of *V. natans.*

| Number | Genera | Orders | Classes | Phylum | References |
| --- | --- | --- | --- | --- | --- |
| 1 | Aeromonas | Aeromonadales | Gammaproteobacteria | Proteobacteria | Morón-López et al., 2024 |
| 2 | Bacillus* | Bacillales | Bacilli | Firmicutes | Morón-López et al., 2024 |
| 3 | Bdellovibrio | Bdellovibrionales | Bdellovibrionia | Bdellovibrionota | Deng et al., 2010 |
| 4 | Brevibacillus | Bacillales | Bacilli | Firmicutes | Morón-López et al., 2024 |
| 5 | Brevundimonas | Caulobacterales | Alphaproteobacteria | Proteobacteria | Morón-López et al., 2024 |
| 6 | Chitinimonas | Burkholderiales | Gammaproteobacteria | Proteobacteria | Coyne et al., 2022 |
| 7 | Chryseobacterium* | Flavobacteriales | Bacteroidia | Bacteroidota | Morón-López et al., 2024 |
| 8 | Cytophaga | Cytophagales | Bacteroidia | Bacteroidota | Morón-López et al., 2024 |
| 9 | Delftia | Burkholderiales | Gammaproteobacteria | Proteobacteria | Morón-López et al., 2024 |
| 10 | Enterobacter | Enterobacteriales | Gammaproteobacteria | Proteobacteria | Morón-López et al., 2024 |
| 11 | Flavobacterium | Flavobacteriales | Bacteroidia | Bacteroidota | Deng et al., 2010 |
| 12 | Kaistia | Hyphomicrobiales | Alphaproteobacteria | Proteobacteria | Morón-López et al., 2024 |
| 13 | Lysinibacillus | Bacillales | Bacilli | Firmicutes | Deng et al., 2010 |
| 14 | Mesorhizobium | Hyphomicrobiales | Alphaproteobacteria | Proteobacteria | Morón-López et al., 2024 |
| 15 | Paenibacillus | Bacillales | Bacilli | Firmicutes | Morón-López et al., 2024 |
| 16 | Paracoccus | Rhodobacterales | Alphaproteobacteria | Proteobacteria | Coyne et al., 2022 |
| 17 | Pseudomonas* | Pseudomonadales | Gammaproteobacteria | Proteobacteria | Morón-López et al., 2024 |
| 18 | Rhodococcus* | Actinomycetales | Actinobacteria | Actinobacteriota | Morón-López et al., 2024 |
| 19 | Sphingomonas* | Sphingomonadales | Alphaproteobacteria | Proteobacteria | Morón-López et al., 2024 |
| 20 | Staphylococcus | Staphylococcales | Bacilli | Firmicutes | Deng et al., 2010 |
| 21 | Stenotrophomonas* | Xanthomonadales | Gammaproteobacteria | Proteobacteria | Morón-López et al., 2024 |
| 22 | Streptomyces* | Streptomycelates | Actinobacteria | Actinobacteriota | Morón-López et al., 2024 |

* denotes genera reported to exert microcystin degradation capability.


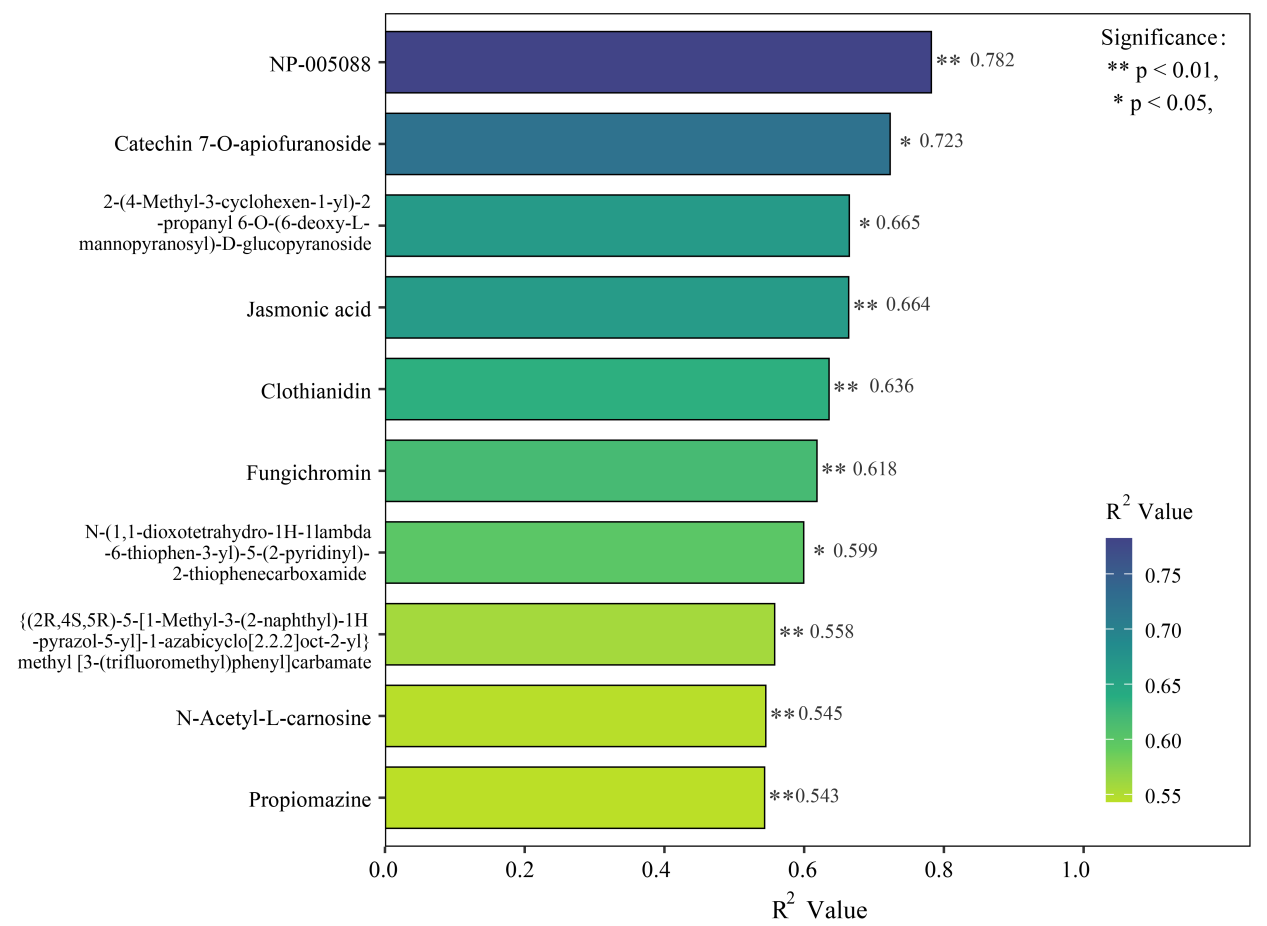


Figure S3 The top 10 metabolites that were significantly correlated with the epiphytic algicidal bacterial community screened by the random forest model.


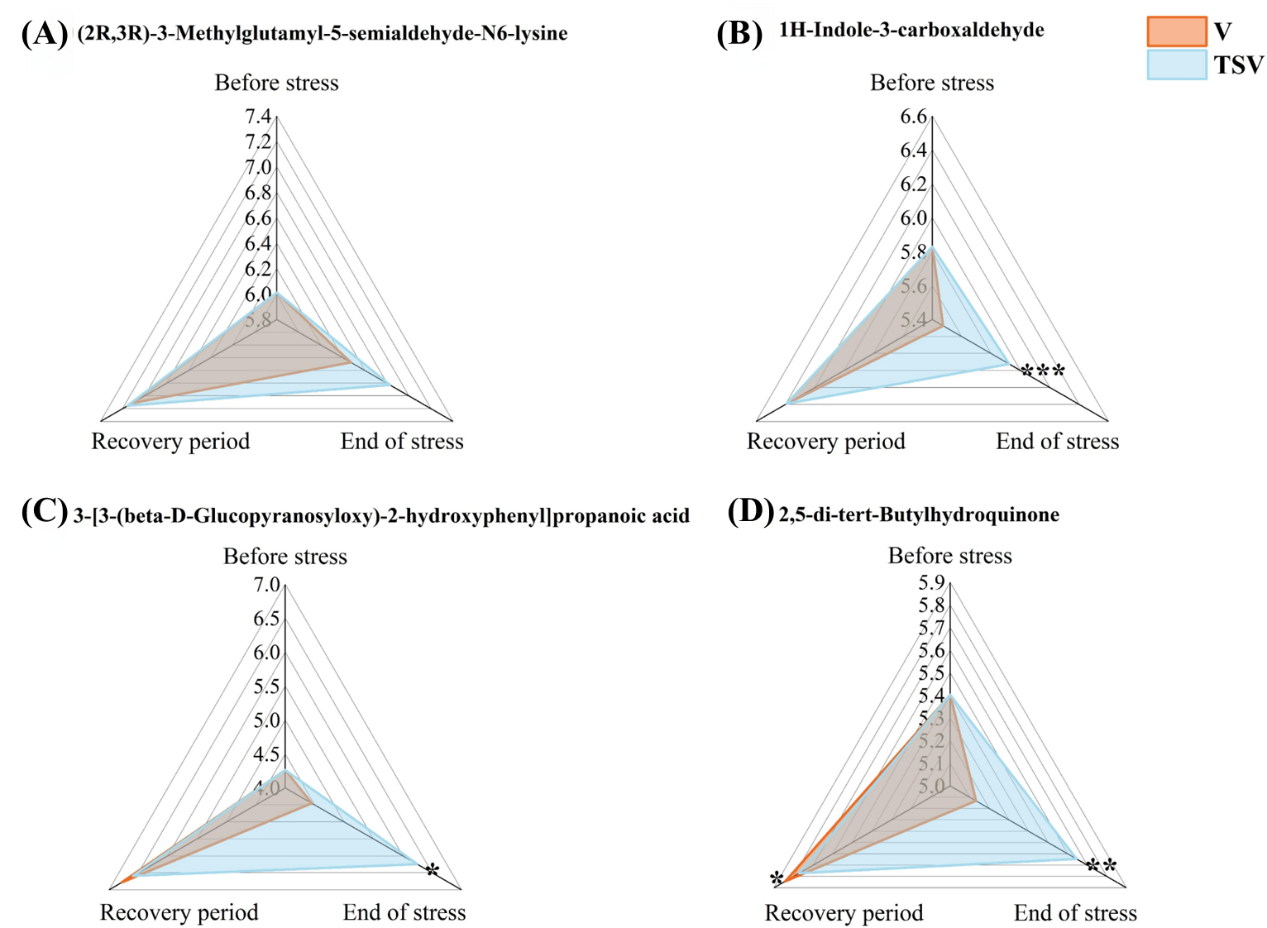


Figure S4 The relative abundance of four potential algicidal metabolites identified from the mixture of *V. natans* and epiphytic microbiomes. Data are means analyzed from three parallel samples. *, ** and ***indicate significant differences between the two groups at p<0.05, p<0.01 and p<0.001, respectively.
